# Supplementary material for: Single-cell multiomics analysis reveals dynamic clonal evolution and targetable phenotypes in acute myeloid leukemia with complex karyotype
Source: Nat Genet. 2024 Nov 25;56(12):2790–803. doi: 10.1038/s41588-024-01999-x (PMC11631769; doi:10.1038/s41588-024-01999-x)
Supplement: Supplementary file 1 — Supplementary Notes 1–9, Figs. 1–12 and references. [file 41588_2024_1999_MOESM1_ESM.pdf]

nature genetics

Supplementary information https://doi.org/10.1038/s41588-024-01999-x

Single-cell multiomics analysis reveals dynamic clonal evolution and targetable phenotypes in acute myeloid leukemia with complex karyotype

In the format provided by the authors and unedited

Supplementary information

Single-cell multi-omics analysis reveals dynamic clonal evolution and targetable phenotypes in acute myeloid leukemia with complex karyotype

Table of content

Supplementary Notes ***3***

Supplementary Note 1. Extended results of clonal dynamics. 3

Supplementary Note 2. Extended results of seismic amplification in patient CK349........................ 5

Supplementary Note 3. Extended results of molecular expression networks in D1922 and CK282 subclones. 6

Supplementary Note 4. Extended results of subclone-specific cell type biases and stemness features. 7

Supplementary Note 5. Extended results of clonal evolution patterns and transcriptomic processes driving CK-AML reconstitution in mice. 7

Supplementary Note 6. Extended results of the *ex vivo* drug screening approach. 8

Supplementary Note 7. Extended results of longitudinal evolution of CK-AML under therapy stress. 8

Supplementary Note 8. Extended discussion of scTRIP vs. bulk genomic analyses. 9

Supplementary Note 9. Extended discussion of subclonal dynamics in primary CK-AMLs. 10

Supplementary Figures ***12***

Supplementary References ***24***

Supplementary Notes

Supplementary Note 1. Extended results of clonal dynamics.

Monoclonal growth:
In the two patients with monoclonal growth, structural variants were shared between all cells (excluding singleton events) affecting three chromosomes in patient HIAML85 and 12 chromosomes in patient CK397 (Fig. 2b, Supplementary Table 3). Both patients harbored inversions at 3q, generating the recurrent oncogenic *RPN1*/*MECOM* fusion described in Fig. 1f. In addition, both patients harbored deletions at chromosome 7 resulting in either monosomy 7 or loss of the almost entire q arm (Fig. 2b, Supplementary Fig. 4a), both of which have been shown to promote disease by cooperating with pre-existing alterations[1,2]. Even though both patients showed dominance of a single subclone at the time of sampling, singleton structural variants present in individual cells were detected in five (HIAML85) or seven cells (CK397) (Supplementary Table 3), suggesting ongoing evolution even in these relatively stable karyotypic contexts. Moreover, one cell in patient CK397 harbored all other clonal structural variants except the deletion at 7q (Fig. 2b, Supplementary Fig. 4a), likely representing a remnant of an out-competed clone. Thus, the acquisition of a strong driver structural variant, such as 3q inversions leading to *MECOM* overexpression, may have favored the selection of a single clone in these patients, generating a largely monoclonal acute myeloid leukemia with complex karyotype (CK-AML).

Linear growth:
The three patients with linear growth (CK295, P9D and HIAML47), were characterized by a step-wise acquisition of structural variants. We identified a set of structural variants in each of the three patients that were present in virtually all cells in these AML samples (Fig. 2c), and thus likely originate from a common precursor AML cell. In all cases, we identified additional structural variants acquired in a step-wise manner, generating the dominant clone at the time of sampling. In patient CK295, 90.2% (37/41 cells) of cells had acquired a duplication of the 8q arm on top of the already present 8p deletion detected in all cells (Fig. 2c, Supplementary Fig. 4b). The dominant clone in patient P9D, making 88.6% of cells (39/44 cells), harbored deletions at 12p and 17p to mid 17q on top of the five clonal structural variants (Fig. 2c). And lastly, in patient HIAML47, 90.1% (82/91 cells) of cells had acquired a duplication at 6p and deletion at 12p as well as focal deletions at 5q and 6q, in addition to the clonal chromosome 8 trisomy and translocation t(15;17)(q14;p12) found in 98.9% (90/91 cells) of cells (Fig. 2c-d, Supplementary Table 3). One cell (1.1%, 1/91 cells) in patient HIAML47 lacked any detectable structural variants (Fig. 2c-d). As this patient progressed from a *JAK2*-mutant myeloproliferative neoplasm (MPN)/chronic myelomonocytic leukemia (CMML) to AML, this cell hints towards the presence of a residual MPN/CMML-related blood cells at the time of CK-AML diagnosis.

Branched polyclonal growth:
From the branched polyclonal samples, D1922 harbored the fewest structural variants, ranging from 4 to 8 structural variants in each cell (Supplementary Table 3). All cells harbored a polyploid chromosome

8 as well as translocation signatures involving the terminal regions of 1p and 6q (Fig. 2e). We further classified the cells into five main subclones, referred to as SC1-SC5 (Fig. 2e). SC1 (47.6%, 30/63 cells) and SC2 (7.9%, 5/63 cells) both harbored a chromosome 5 trisomy with SC2 further carrying a chromosome 20 trisomy. In contrast, SC3 (27.0%, 17/63 cells), SC4 (11.1%, 7/63 cells) and SC5 (6.4%, 4/63 cells) lacked the chromosome 5 trisomy but harbored a chromosome 21 trisomy instead. SC4 additionally carried trisomies of chromosome 16 and 19 whereas SC5 harbored a complex rearrangement at chromosome 6, involving two deletions and two inverted duplications, reminiscent of chromothripsis. Beyond these subclone-defining rearrangements, we identified one cell part of SC2 with a terminal deletion at 4q.

In patient CK349, we identified a set of structural variants affecting eight chromosomes that were present in all cells (Fig. 2e). We further classified the cells into three main subclones, referred to as SC1, SC2 and SC3 characterized by 16-63 structural variants in each cell (Fig. 2e, Supplementary Table 3). SC1 (89%; 81/91 cells) represented the largest clone and harbored a chromosome 8 trisomy. Chromosome 13 showed signs of active mutational processes in multiple cells of SC1, resulting in additional somatic gains or losses of terminal chromosome regions (duplication: 3/81 cells; deletion: 4/81 cells), whole-chromosome aneuploidies (gain 3/81 cells; loss 4/81 cells) and complex rearrangements involving serially acquired structural variants (2/81 cells) (Extended Data Fig. 2a-b) – together indicating clear ongoing instability for this chromosome. In SC2 (5.5%; 5/91 cells) and SC3 (5.5%; 5/91 cells) we identified a distinct set of rearrangements also affecting chromosome 13 but different from the structural variants described above. These consisted of two duplications (10/10 cells) juxtaposed next to a nonamplified (7/10 cells) or deleted region (3/10 cells), spanning the whole chromosome 13 (Extended Data Fig. 2a-b). SC3 harbored an additional set of structural variants at chromosome 11, resulting in the characteristic wave-like copy-number profiles of seismic amplifications described in Figure 2f-h. As a consequence, this subclone displayed distinctively high levels of intra-patient heterogeneity compared to SC1 and SC2. Beyond this, all CK349 cells harbored deleted segments at chromosome 17, differing in lengths (Extended Data Fig. 4c-d). We identified 15 non-overlapping breakpoints that preceded the deleted segments with most of the distinct breakpoints shared only between few cells (Extended Data Fig. 4d). While the non-overlapping breakpoints indicate continued chromosomal instability on this chromosome, SC3 cells were enriched in one distinct deleted segment (Extended Data Fig. 4d). This hints towards stabilization of a chromosomal intermediate in a subset of SC3 cells in CK349. Additionally, we identified singleton structural variants present in individual cells.

Amongst all the CK-AMLs, CK282 showed the most abundant subclone diversity, represented by five distinct subclones characterized by 6-59 structural variants in each cell (Fig. 2e, Supplementary Table 3). We identified an inversion at 3q present in all cells and an additional set of structural variants

affecting nine chromosomes present in all but a small set of cells, referred to as SC5 (3.95%, 3/76 cells). Three subclones, referred to as SC1 (19.7%, 15/76 cells), SC2 (5.3%, 4/76 cells), and SC3 (44.7%, 34/76 cells), showed a high level of genetic similarity, with the exception of structural variants identified on chromosomes 8 and 20. Unique to SC2, we identified two inverted duplications, three deletions and one larger inverted duplication on chromosome 8 (Extended Data Fig. 3b), suggesting the occurrence of breakage-fusion-bridge (BFB) cycles with additional islands of loss of heterozygosity. In SC1, SC2, and SC3, chromosome 20 displayed a classical BFB event with the typical inverted duplication and adjacent terminal deletion structural variant signature arising on the same haplotype (Extended Data Fig. 4a-b). While the BFB was a shared event, the length of the terminal deletion varied between SC1-SC3. SC1 and SC2 showed 8 distinct breakpoints for the 20q terminal deletion whereas all cells in SC3 presented a uniform breakpoint (Extended Data Fig. 4b). This suggests the presence of unstable chromosomal intermediates in SC1 and SC2, which may have become stabilized in SC3. SC4 (25.0%, 19/76 cells) lacked rearrangements on chromosome 20 but displayed several unique structural variants, including three duplications on chromosomes 9, 12 and 18, and one inversion on chromosome 17, respectively (Fig. 2e, Supplementary Table 3). By comparison, SC5 (3.95%, 3/76 cells) markedly differed from all other subclones, and harbored a distinct and much smaller structural variant set and lacked complex rearrangements abundant in SC1–SC4 almost entirely (Fig. 2e, Supplementary Table 3). Beyond this, we identified singleton structural variants present in individual cells.

Collectively, our data showcase that multiple clones can expand simultaneously in CK-AML. This includes comparatively simple subclones with stepwise acquisition of additional lesions next to genetically highly complex ones with intra-patient branched and parallel evolution of highly distinct subclones originating from a common leukemic precursor cell.

Supplementary Note 2. Extended results of seismic amplification in patient CK349.

Seismic amplifications have been proposed to represent a multistep rearrangement process initiated in most cases via a chromothripsis event[5]. This leads to the formation of circular extrachromosomal DNA (ecDNA) that subsequently undergoes repeated rounds of circular recombination, followed by a potential stabilization step, e.g. via linearization, and which ultimately results in a seismic-wave like copy-number pattern in bulk sequence data[5]. The dynamics of seismic amplification events are presently unexplored, and, to the best of our knowledge, the process of seismic amplification has previously not been analyzed in single cells. Leveraging the single-cell structural variant detection resolution of Strand-seq, we characterized these chromosome 11 events in detail aiming to reconstruct the series of rearrangements present within the CK349 subclone SC3. In the five cells of the subclone, all chromosome 11 structural variants mapped to the same haplotype (H1; Fig. 2f, Extended Data Fig. 5b), consistent with a single chromosomal homolog initiating the chromosomal instability process. Furthermore, our analysis revealed a step-wise acquisition of amplifications with cell-specific copy-

number statuses on chromosome 11. For instance, we identified two cells showing intermediate levels of amplification mostly localized to the q arm as well as three cells with excessive and more widespread amplifications spanning both p and q arms (Fig. 2f, Extended Data Fig. 5a-b), suggesting a potential difference in the number of circular recombination rounds between individual cells. All cells with seismic amplifications shared two common deletions at the q arm: (1) a deletion located between two invertedly duplicated segments; and (2) a terminal deletion adjacent to an inverted duplication segment (Fig. 2f, Extended Data Fig. 5a-b). These data indicate that a fold-back inversion at 11q was potentially triggered by a telomeric loss, and this served as an initiator of the complex rearrangement, leading to circularization and repeated recombination of the affected chromosome 11 homolog (Fig. 2g). To verify the presence of ecDNA, we performed multiplex fluorescence in situ hybridization (M-FISH) analysis of a patient-derived xenograft (PDX) sample generated from CK349, which revealed a large ring chromosome containing several copies of segments from 11p and 11q (Fig. 2h). These data represent, to our knowledge, the first report and comprehensive characterization of a seismic amplification in single cells, revealing that all cells affected through this rearrangement process differ from one another.

Supplementary Note 3. Extended results of molecular expression networks in D1922 and CK282 subclones.

In patient D1922, cells in SC1 and SC2 stemming from the same branch showed enrichment of oxidative phosphorylation and glycolysis-associated gene signatures, whereas SC3, SC4 and SC5 forming the second branch were enriched in hedgehog signaling and allograft rejection-associated gene sets (Supplementary Fig. 7a). Similarly, in patient CK282, genes involved in mitochondrial complex V (*ATP5MF*, *ATP5MG*, *ATP5MD*) showed upregulation in cells from SC1, SC2 and SC3 that stemmed from the same branch (Fig. 3b, Supplementary Table 5). This was also reflected in enrichment of oxidative phosphorylation gene set (Fig. 3c). In contrast, SC4 and SC5 each formed their own branches and upregulated distinct pathways, including TNF-α signaling via NF-κB in SC4 and interferon gamma response in SC5 (Supplementary Fig. 7b). These data suggest that subclones within the same patient, but with different genetic profiles show activation of distinct molecular pathways to leverage growth in a competitive setting.

While cells within the same subclone or subclonal branch shared epigenetic and transcriptomic programs, cells within each subclone also showed unique gene activity programs as well as heterogeneity within a subclone. This was as evident in the distribution of cells along the low-dimensional subspace, with cells of different genotypes occasionally sharing similar phenotypic states (Fig. 3a), supporting the hypothesis that a leukemic cell´s phenotype is determined by both genetic and non-genetic factors[6].

Supplementary Note 4. Extended results of subclone-specific cell type biases and stemness features.

In addition to pathway dysregulation, cell type composition and stemness vary between AML patients and have been shown to have prognostic significance[7]. Yet, whether these characteristics are specific to subclones, and thus potentially influenced by the AML's subclonal structure, has remained unclear. To assess cell type abundances in our cohort, we performed cell-typing of each CK-AML Strand-seq library using a scMNase-seq-based cell-type classifier derived from index-sorted healthy bone marrow CD34+ cells[8] (Supplementary Table 7). We observed a high abundance of hematopoietic stem cell (HSC)-like and/or megakaryocyte–erythroid progenitor (MEP)-like cells in 6 of 8 CK-AML patients independent of the subclonal growth patterns (Supplementary Fig. 8a), consistent with previous studies[9-11]. Moreover, in 4 of 6 patients with linear and branched polyclonal growth at least one subclone demonstrated a strong bias towards a specific cell type, accounting for ≥ 50% of cells in that subclone (Supplementary Fig. 8b). These lineage-biased subclones included, for instance, SC1 in patient P9D enriched in HSC-like cells, SC2 in patient CK282 enriched in common myeloid progenitor (CMP)-like cells, and SC3 in patient CK295 enriched in granulocyte-monocyte progenitor (GMP)-like cells (Supplementary Fig. 8b). Next, we leveraged the somatic copy-number alteration recalling from CITE-seq data to assess transcriptomic stemness features across patients and subclones. While high leukemic stem cell (LSC) scores[12] generally showed association with primitive cell types and high structural variant burden (Supplementary Fig. 8c-e), they were not restricted to one cell type and showed extensive heterogeneity within subclones (Supplementary Fig. 8f). As known for clonal AML driver structural variants[10,13], our data suggest that CK-AML, while heterogeneous, can show subclone-specific cell type biases. These data should motivate future functional studies that aim to interrogate the biological processes underlying cell type bias and its association with genetics.

Supplementary Note 5. Extended results of clonal evolution patterns and transcriptomic processes driving CK-AML reconstitution in mice.

To explore the longitudinal clonal dynamics of CK-AML cells and characterize their associated disease propagating potential, we established PDX models for five patients (Supplementary Table 1) and analyzed the engrafting cells using scNOVA (Fig. 4a). This revealed two engraftment patterns in PDX: (a) engraftment of the dominant clone (HIAML85, HIAML47) or (b) engraftment of a minor subclone (CK282, CK349, CK397) (Fig. 4b-c). In patients HIAML85 and HIAML47, major subclones re-established AML in mice (SC1 in HIAML85 and SC3 in HIAML47), constituting 95.2% (59/62 cells) and 100% (54/54 cells) of the respective PDX cells. In contrast, in patients CK282, CK349 and CK397, minor subclones re-established AML in mice (SC2 in CK282, SC3 in CK349 and SC3 in CK397; Fig. 4c). In CK282 and CK349 these subclones also showed the highest LSC scores, structural variant burden and karyotype heterogeneity with ongoing chromosomal instability in the primary sample (Fig. 2e-f, Supplementary Fig. 8f). The relative size of the minor engraftment-driving subclones increased from 5.3% (4/76 cells) and 5.5% (5/91 cells) in these two patient samples at diagnosis to 97.8% (45/46 cells)

and 97.5% (39/40 cells) in the respective PDXs (Fig. 4c). In patient CK397, the PDX cells consisted of two distinct subclones with 11.1% of the engrafted cells (4/36 cells) bearing the structural variants of the dominant AML clone, whereas 88.9% of the engrafted cells (32/36 cells) carried additional structural variants. Only one cell at diagnosis resembled the latter cells forming the major clone in the PDX (Fig. 4c), indicating previously undetected clonal evolution expanding in the mouse.

In order to examine the transcriptomic processes facilitating the engraftment and proliferation of a particular subclone, we traced in three CK-AMLs with linear or branched growth patterns at diagnosis (CK282, CK349, and HIAML47) the engrafted cells within the PDX models back to the subclones present in the primary patient samples. We identified 15 genes that were upregulated in the engraftment-driving LSCs in all three CK-AML samples (FDR < 0.05 and logFC > 0.1), including *MTSS1*, *PHACTR1* and *LSP1* all involved in cytoskeletal organization and cell adhesion (Supplementary Fig. 9a, Supplementary Table 8). Generally, the engraftment-driving cells shared transcriptomic programs involved in cell growth, proliferation and oxidative phosphorylation, whereas non-engrafting cells were enriched in gene sets associated with inflammation (Supplementary Fig. 9b). Collectively, we identified different clonal evolution fates and patterns during CK-AML reconstitution in mice with increased homing and cell growth properties likely contributing to this reconstitution.

Supplementary Note 6. Extended results of the *ex vivo* drug screening approach.

To assess drug-response profiles between subclones, we included three patient samples that showed linear or branched polyclonal growth patterns at diagnosis (HIAML47, CK349 and CK282) for follow-up studies. First, we developed antibody panels tailored to capture the distinct subclones present in each sample, leveraging the differentially expressed cell surface proteins from our CITE-seq data (Fig. 5a-b, Supplementary Fig. 11a-e). Second, we measured the *ex vivo* drug response by culturing the bulk CK-AML cells in the presence of up to 12 different clinical drugs that included standard chemotherapy regimens as well as novel compounds, such as BH3 mimetics (Fig. 5a, Supplementary Table 9), which we and others have shown to be able to target LSCs[14-18]. Third, we stained the diagnosis samples with their unique antibody combinations to assess the drug-response profiles of each subclone by flow cytometry (Fig. 5a, Supplementary Fig. 11f).

Supplementary Note 7. Extended results of longitudinal evolution of CK-AML under therapy stress.

Patient P5 achieved complete remission after treatment with induction chemotherapy consisting of cytarabine, daunorubicin and etoposide but relapsed 167 days later (Fig. 6a). At diagnosis the patient showed a branched polyclonal growth pattern with the presence of five distinct subclones (Fig. 6b). Upon relapse, only one subclone remained that closely resembled diagnosis subclone SC1 (Fig. 6b). The relapse cells had additionally acquired a novel complex rearrangement on chromosome 6 which was present in 98% of the cells (53/54 cells) and showed ongoing instability (Extended Data Fig. 10a).

The rearrangement included terminal deletions at 6p and 6q surrounded by inverted duplications with varying copy numbers, reminiscent of chromothripsis and manifesting as a marker chromosome (Fig. 6c, Supplementary Table 1). These structural variants were distinct from the set of complex rearrangements affecting the same chromosome in SC5 at diagnosis, suggesting a role of specific chromosome 6 structural variants in therapy resistance and relapse occurrence. Relapse cells also upregulated MYC targets and oxidative phosphorylation gene signatures compared to treatment naïve cells at diagnosis (Fig. 6e-g, Extended Data Fig. 10d, Supplementary Table 10), with oxidative phosphorylation already upregulated in SC1 cells at diagnosis (Supplementary Fig. 7a). MYC targets also remained enriched in relapse cells, even when comparing the SC1-derived relapse cells only with SC1 cell from diagnosis (Extended Data Fig. 10d).

Patient P9 was treated first-line with the BCL-2 inhibitor venetoclax together with azacytidine, but was refractory to this treatment (Fig. 7a, Supplementary Fig. 10). At diagnosis, P9 CK-AML cells showed a linear growth pattern with three subclones detected (Fig. 7b). We analyzed the day 12 post-treatment sample where two distinct subclones persisted (Fig. 7b). 85.7% of the refractory cells (18/21 cells) resembled SC3 at diagnosis, harboring the same structural variant profile, including a terminal deletion at 12p and larger deletion at chromosome 17 overlapping, for example *TP53* and *NF1*, without novel *de novo* structural variants (Fig. 7b-c, Extended Data Fig. 10e). The remaining 14.3% of cells (3/21 cells) resembled SC1 at diagnosis and lacked SC3-specific structural variants on chromosome 12 and 17 (Fig. 7b-c). These SC1-derived cells had gained a novel 5Mb focal deletion on chromosome 17q, a region containing the tumor suppressor gene *NF1* (Fig. 7c). Loss of *NF1* is known to cause Ras pathway activation which has further been linked to venetoclax resistance[19]. While *NF1* showed reduced expression specifically in the SC1-derived cells in the refractory sample (Fig. 7e-f), *NF1* also showed increased nucleosome occupancy in SC1 cells already at diagnosis (Supplementary Table 6), suggesting a combination of genetic-and epigenetic mechanisms responsible for the failure of treatment response in this subclone.

Supplementary Note 8. Extended discussion of scTRIP vs. bulk genomic analyses.

Strand-seq with scTRIP structural variant discovery framework provides unmatched resolution for reconstruction of subclonal architectures in CK-AML. Yet, plate-based single-cell methods have an inherent limitation with regard to cell numbers[20], and Strand-seq only captures structural variants of at least 200 kb in size[4,21]. Thus, the extent of genetic heterogeneity reported in this study could still be an underestimate as small structural variants and subclones may have been missed. This highlights an important area for future single-cell technology development. Regarding the sensitivity of detecting structural variants at a global level, Sanders et al. showed that scTRIP structural variant calling was able to confirm 100% and 89% of the tested structural variants present by whole genome sequencing (WGS)[4]. Jeong et al. also verified all somatic structural variants detected in a lymphoblastoid cell line using

scTRIP with deep WGS data[22]. However, compared to bulk analyses, scTRIP is limited to the detection of structural variants larger than 200kb in size[4]. While this impedes the detection of small structural variants, single nucleotide variants and indels, 80% of all structural variant drivers in cancer genomes tend to be at least 200 kb in size[22]. Thus, the majority of functionally relevant structural variants in our cohort were likely caught.

Compared to single cell sequencing, standard 30x–50x WGS is not able to capture both low-frequency variants and clonal complexity, as demonstrated in several prior works[23]. Cell fractions >30% are generally considered amenable to bulk WGS-based structural variant calling[24], whereas scTRIP can detect structural variants at cell fraction levels significantly below that – even cell fractions of around 1.5%[4]. Thus, extremely small subclones with a clonal fraction less than 1.5% may not have been detected in our study. Yet, subclones with clonal fractions ranging from 2% to 10% are effectively identifiable using our technique, while practically unattainable with bulk tumor sequencing. Here, all CK-AMLs with polyclonal growth at initial sampling (6/8 samples) harbored subclones that made <30% of the cells (Fig. 2c and e), highlighting that the ability to detect subclones is important for the majority of CK-AML samples. Moreover, different CK-AML subclones within the same sample can have different sets of structural variants, evident in particular in the samples with branched polyclonal growth (Fig. 2e). For example, SC3 in patient CK349 (5.5%, 5/91 cells) harbored a seismic amplification at chromosome 11 but lacked the chromosome 8 trisomy present in the majority of the cells (SC1; 89%, 81/91 cells) (Fig. 2e). Using bulk WGS for detecting structural variants, the spectrum of chromosome 11 rearrangements would likely have remained obscured and the pattern of co-occurrence of chromosome 11 rearrangements and chromosome 8 trisomy virtually impossible to determine. The driving force behind the clonal relapse in patient CK349 was SC3 (Fig. 2e, 4f-g), further underscoring the necessity of differentiating among subclones for biological and clinical insights, often not achievable through bulk WGS analysis.

Supplementary Note 9. Extended discussion of subclonal dynamics in primary CK-AMLs.

Our data provide intriguing insight into patient-specific subclonal dynamics in primary CK-AMLs pre- and post-treatment as well as functional analysis of LSCs by analysis of PDX models. In diagnosis/salvage samples, five patients supported the concept of selective sweeps[25], with monoclonal or linear growth resulting in the dominance of a single clone (Fig. 2a-c). We also detected branched polyclonal growth, whereby multiple subclones were present in parallel (Fig. 2a, e), greatly extending original findings revealed by metaphase karyotyping of a large AML cohort[26]. As evident in our temporal analysis, individual cases of AML may not follow a single mode of tumor growth but different modes can operate in parallel during different stages of leukemogenesis[27]. CK-AML reconstitution in mice showed engraftment of LSCs derived from either a major or a minor subclone of a patient, resulting in either case in the strong dominance of that single subclone reconstituting the CK-AML in the mice

(Fig. 4b-c). This was also observed in the two relapse cases (Fig. 4f-g, 6b). Such monoclonal sweeps are also common in metastasis formation in solid cancers[28,29]. Conversely, treatment pressure in CK-AML patients may not always result in outgrowth of a single clone[30], as revealed in a treatment refractory sample in which two venetoclax/azacytidine resistant subclones persisted (Fig. 7b). Importantly, we also observed ongoing chromosomal instability affecting specific chromosomes in individual CK-AML cells within rather stable subclones both at diagnosis and under selection pressure (Fig. 6c, Supplementary Fig. 5a). Ongoing genomic instability in single cells included capturing unstable chromosome intermediates as a result of chromothripsis[31] and prolonged BFB cycles[5,32,33], likely enabled by dysfunctional p53, which can promote genome plasticity and the emergence of drug-resistant clones[34]. This was indeed seen in the two patients that relapsed after standard chemotherapy (Fig. 4f-g, 6a-c), highlighting genome plasticity in CK-AML cells as a strategy to escape treatment.

Supplementary Figures

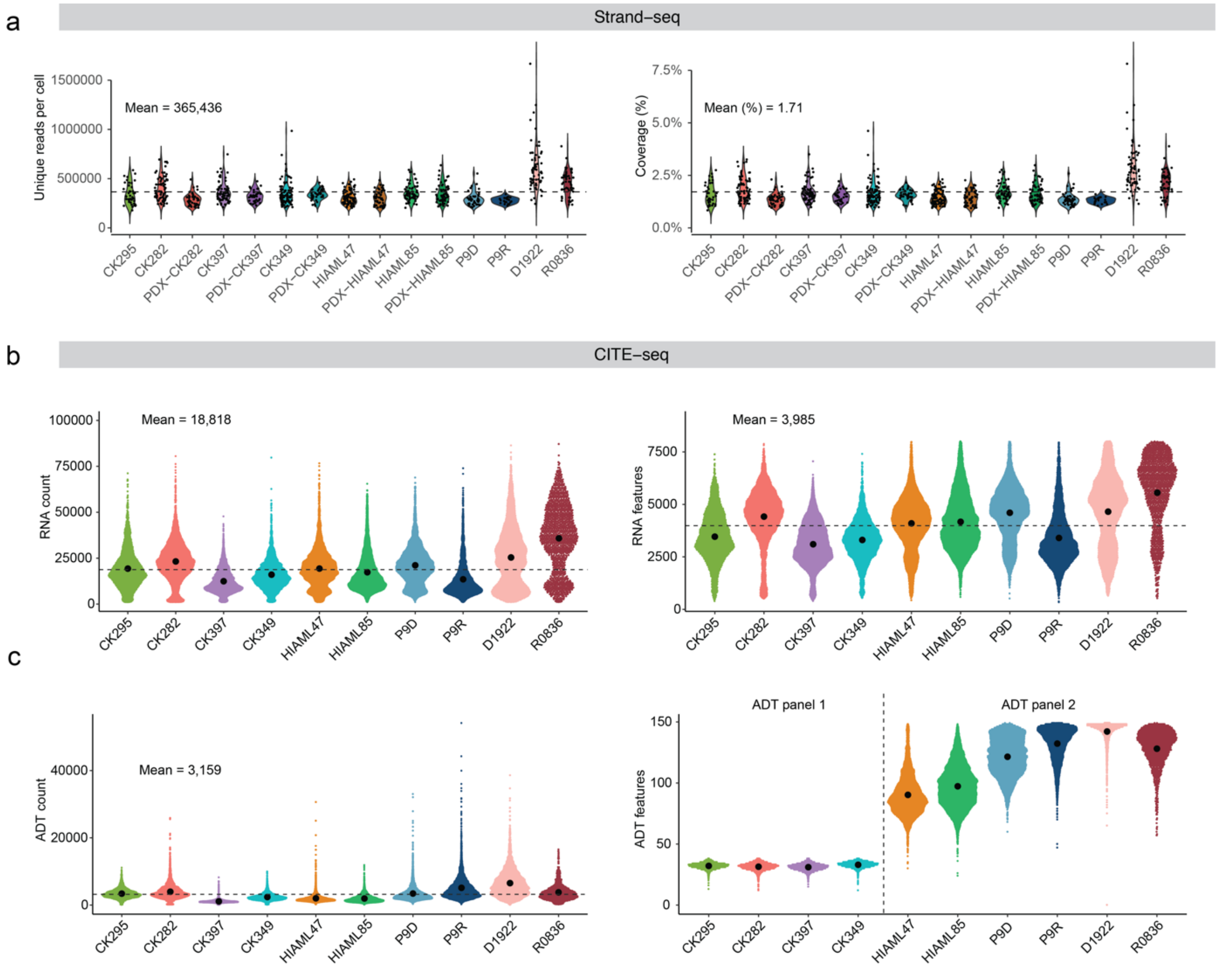

Supplementary Figure 1. Strand-seq and CITE-seq statistics. a Number of unique reads detected (left) and coverage (right) per single cell passing filter per sample (n = 21– 91). **b** Gene expression statistics in CITE-seq data. Number of RNA molecules (left) and number of unique genes (right) detected per single cell passing filter per sample (n = 1,314– 6,394 cells). **c** Cell surface expression statistics in CITE-seq data. Number of antibody-derived tag (ADT) molecules (left) and unique ADTs (right) detected per single cell passing filter per sample (n = 1,314– 6,394 cells).
Dashed lines show the mean and beeswarm plots show the 95% confidence interval for the mean.

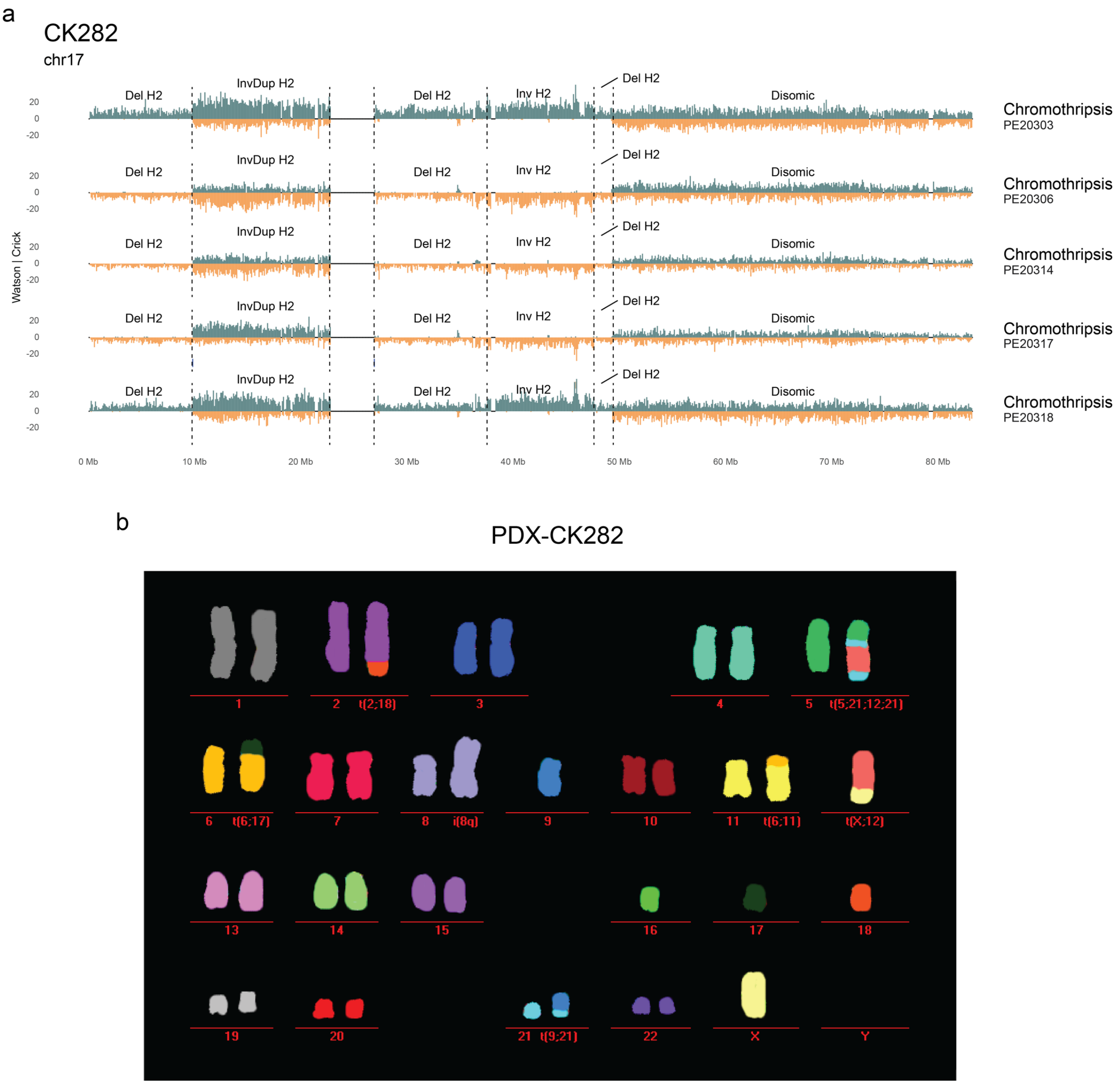

Supplementary Figure 2. Complex structural variant formation in CK282 a Strand-specific read depth of representative single cells from CK282 showing clustered deletions (Dels), inverted duplications (InvDels) and inversions (Invs) along a single homolog (H2) at chromosome (chr) 17, resulting from clonal chromothripsis. Reads denoting somatic structural variants, discovered using scTRIP, mapped to the Watson (orange) or Crick (green) strand. Grey: single cell IDs. **b** Multiplex fluorescence *in situ* hybridization (M-FISH) of a cell from the patient-derived xenograft (PDX) of CK282.

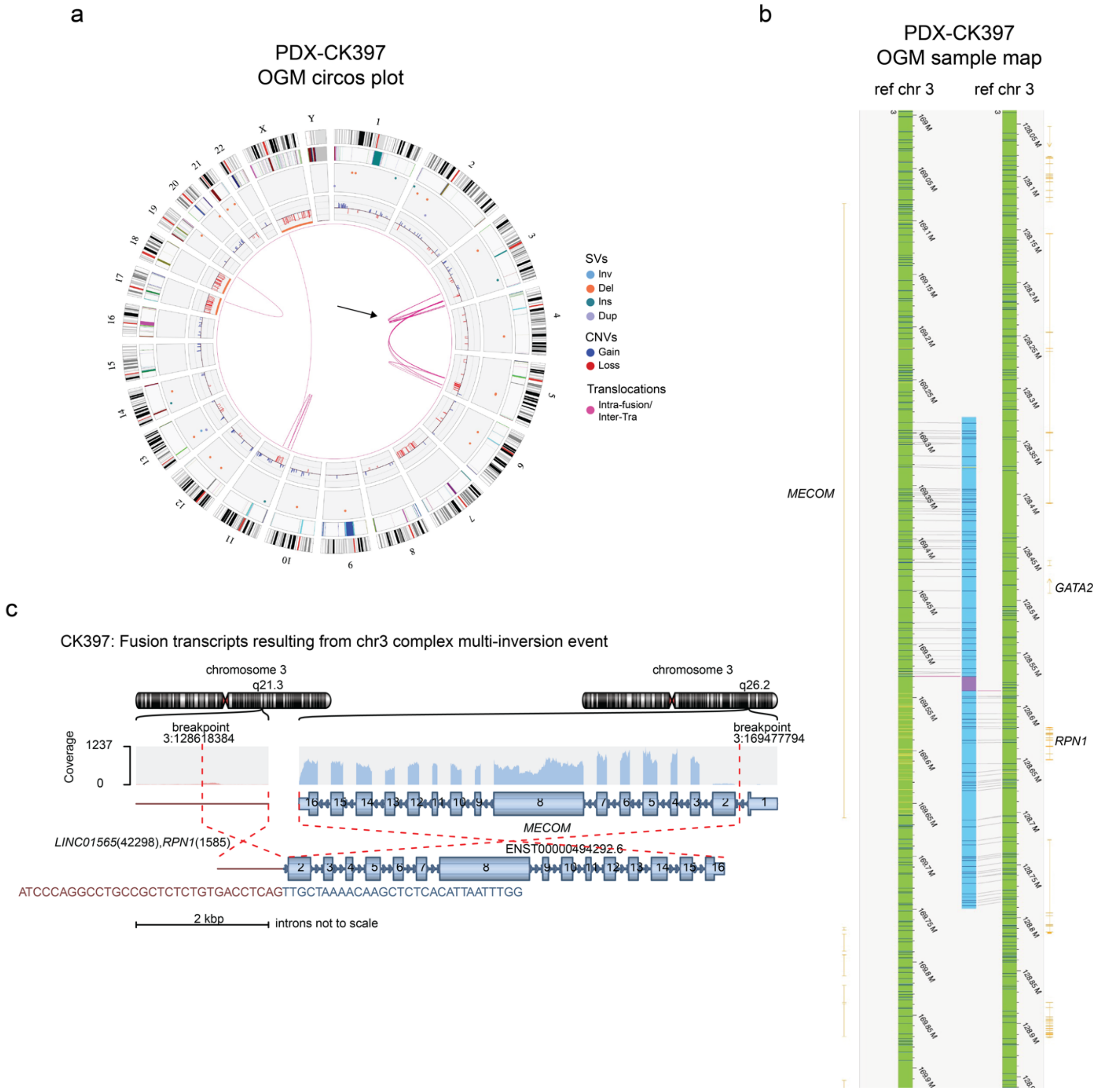

Supplementary Figure 3. Intra-chromosomal rearrangements at 3q in CK397. a Circos plot illustrating the inter- and intra-chromosomal rearrangements assessed by optical genome mapping (OGM) from patient-derived-xenograft (PDX) of CK397. Chromosomes (outside of the circular plot) and chromosomal rearrangements are shown as arcs connecting the two relevant genomic regions in the middle. Arrow indicates the intra-chromosomal rearrangement at chromosome 3. **b** Fine mapping of DNA segments by OGM showing an intra-chromosomal rearrangement between segments from 3q resulting in *RPN1*/*MECOM* fusion in engrafted cells from CK397. The blue bar shows the alignment of the sample's consensus map with the references (ref) shown as green bars. **c** Fusion transcripts between *MECOM* and the intergenic region of *RPN1* confirming the intra-chromosomal rearrangement at 3q. Shown are the fusion partners, their orientation, the retained exons in the fusion transcript, the expression of the exons and the excerpt sequence around the breakpoint. SV: Structural variant, Inv: inversion, Del: Deletion, Ins: Insertion, Dup: Duplication, Tra: Translocation, Chr: Chromosome.

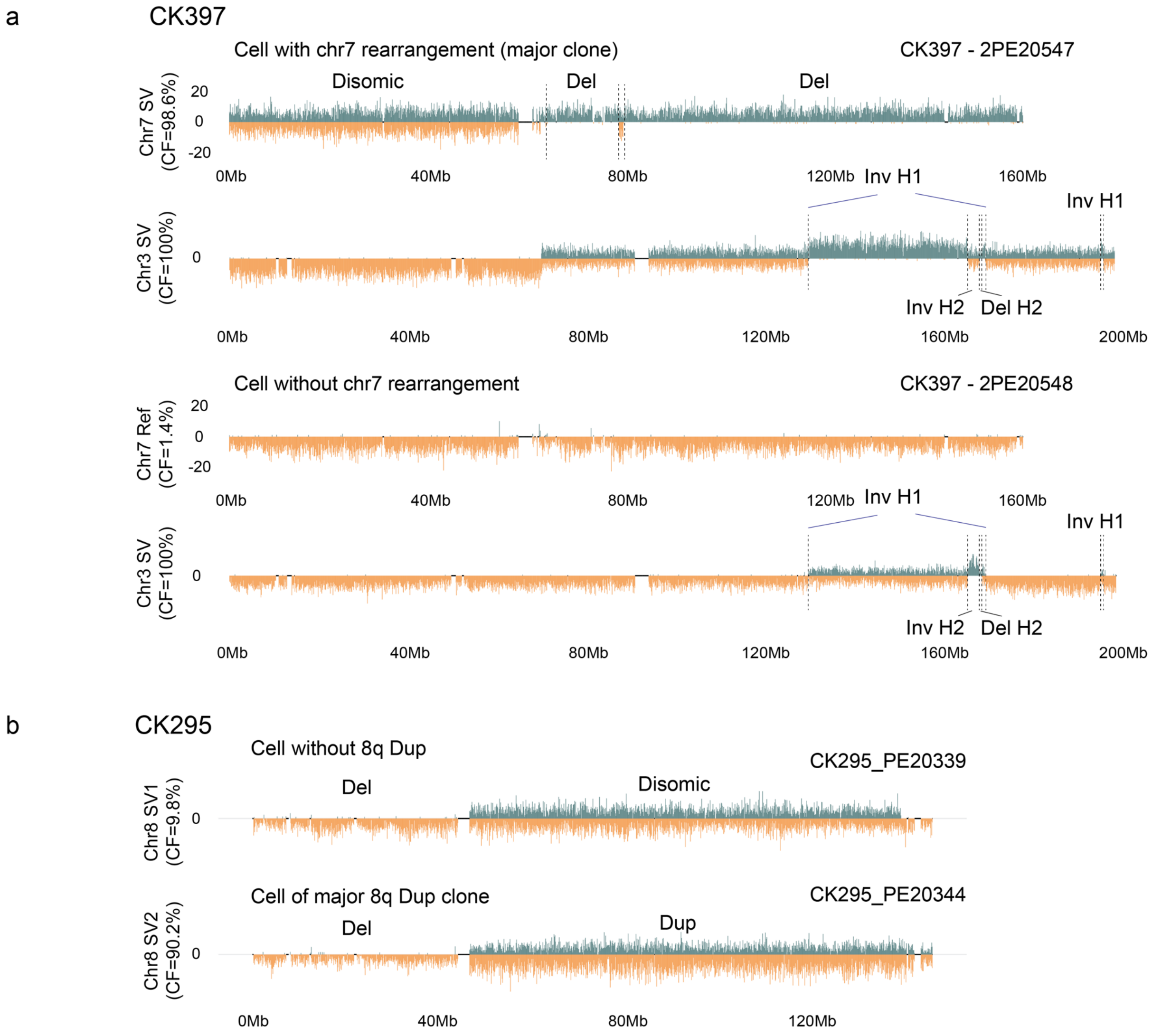

Supplementary Figure 4. Subclonal evolution in CK397 and CK295. **a** Chromosome 7 and chromosome 3 in two representative single cells from CK397 portraying two subclones identified in CK397. Top plot shows a cell from the major subclone with a large 7q deletion spanning almost the entire arm and a rearranged chromosome 3, represented by 69 cells. Bottom plot shows a cell from the minor subclone with a normal chromosome 7 (reference) and a rearranged chromosome 3, represented by a single cell. **b** Chromosome 8 in two representative single cells from CK295 portraying two subclones identified in CK295. Top plot shows a cell from the minor subclone with an 8p deletion and normal 8q arm, represented by four cells. Bottom plot shows a cell from the major clone with an 8p deletion and 8q duplication, represented by 37 cells. Reads denoting somatic structural variants, discovered using scTRIP, mapped to the W (Watson, orange) or C (Crick, green) strand. Grey: single cell IDs. Chr: Chromosome, CF: Cell fraction, SV: Structural variant, Del: Deletion, Inv: Inversion, H: Homolog, Dup: Duplication.

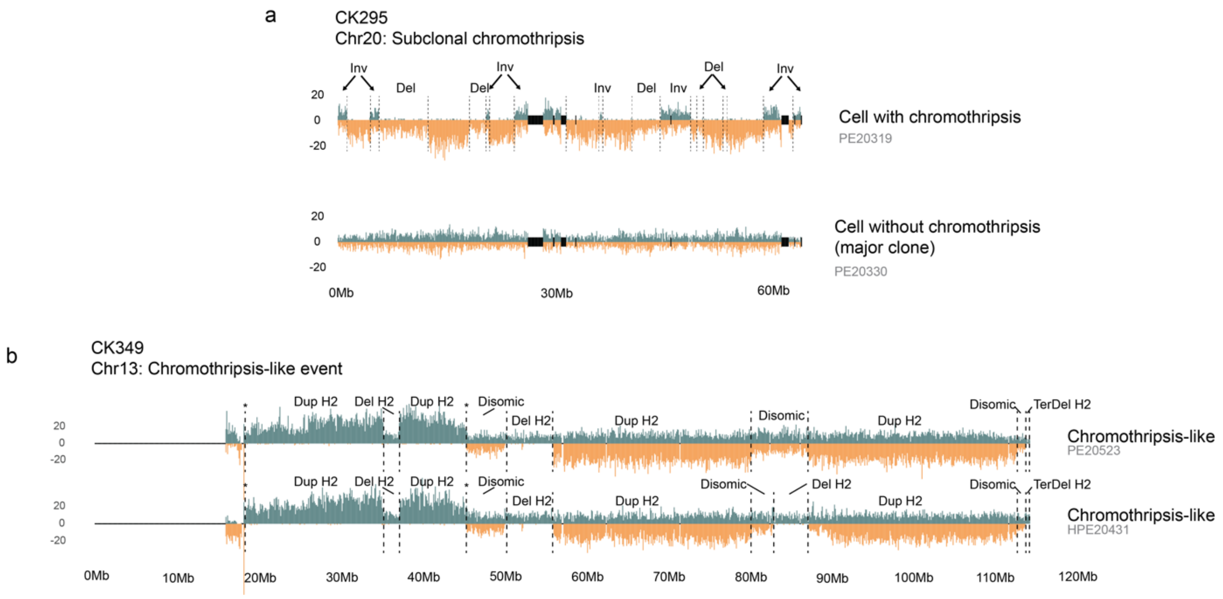

Supplementary Figure 5. Complex genomic rearrangements in CK295 and CK349. a Strand-specific read depth of an example CK295 cell showing clustered deletions and inversions along a single homolog at chromosome 20, resulting from chromothripsis (top), compared to a cell without chromothripsis (bottom). **b** Strand-specific read depth of two representative single cells from CK349 with chromothripsis-like but serially acquired rearrangements at chromosome 13, comprising duplications and deletions. (*) indicates switch in strand. Grey: single cell IDs. Del: Deletion, Inv: Inversion, H: Haplotype, Dup: Duplication, Ter: Terminal.

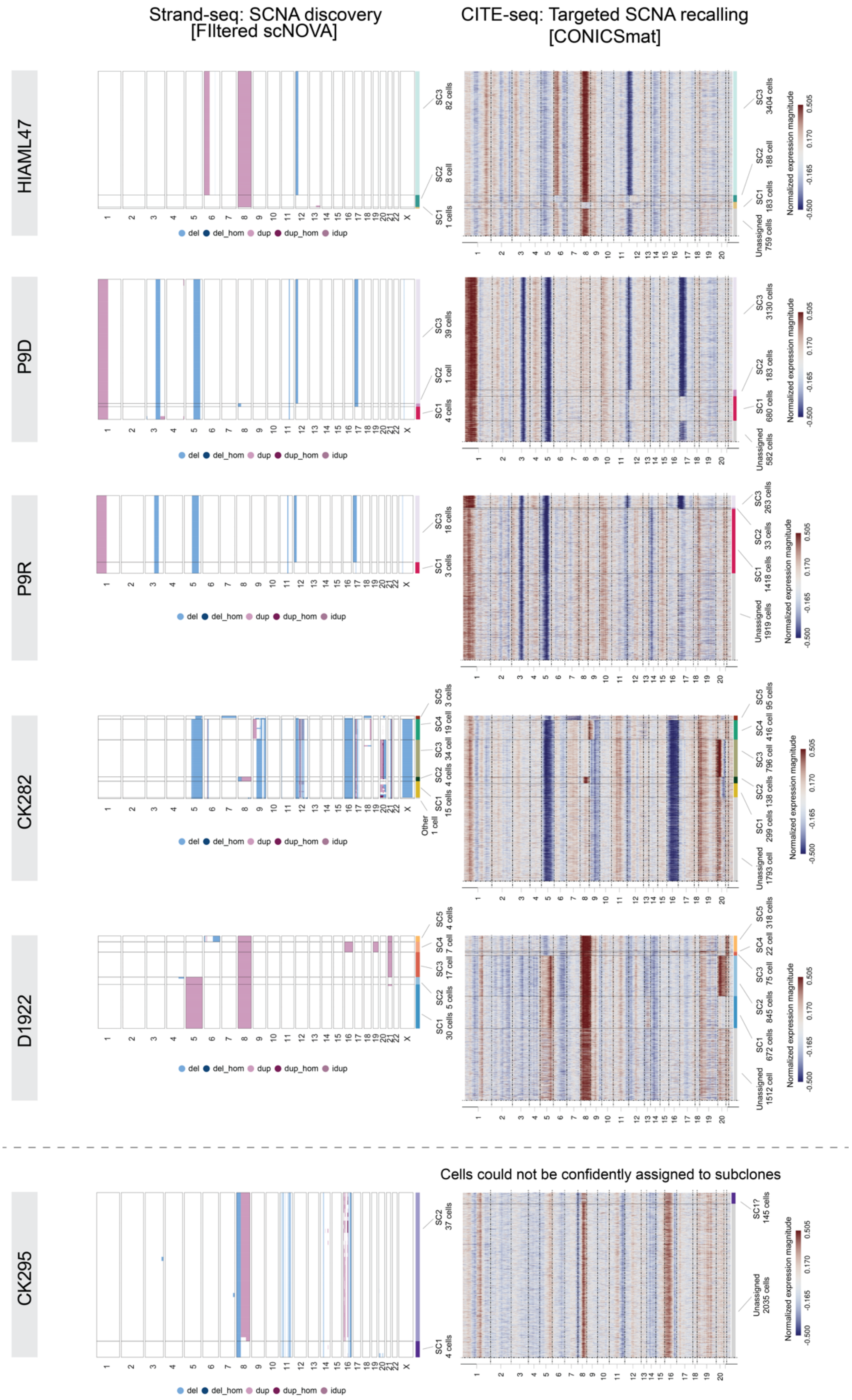

Supplementary Figure 6. Subclonal heterogeneity between Strand-seq and CITE-seq-based assessment. Somatic copy number alteration (SCNA) discovery based on scNOVA from Strand-seq data (left) and targeted SCNA recalling based on CONICSmat from CITE-seq data (right). Subclone assignments and corresponding cell numbers are shown on the right of each heatmap. In patient CK295, cells could not be confidently assigned to subclones based on likelihood ratio test adjusted p-value < 0.01, Bayes Information Criterion (BIC) > 200 and posterior probability cutoff criteria of 0.8. See Extended Data Fig. 6b for patient CK349. del: deletion, hom: homologous, dup: duplication, idup: inverted duplication.

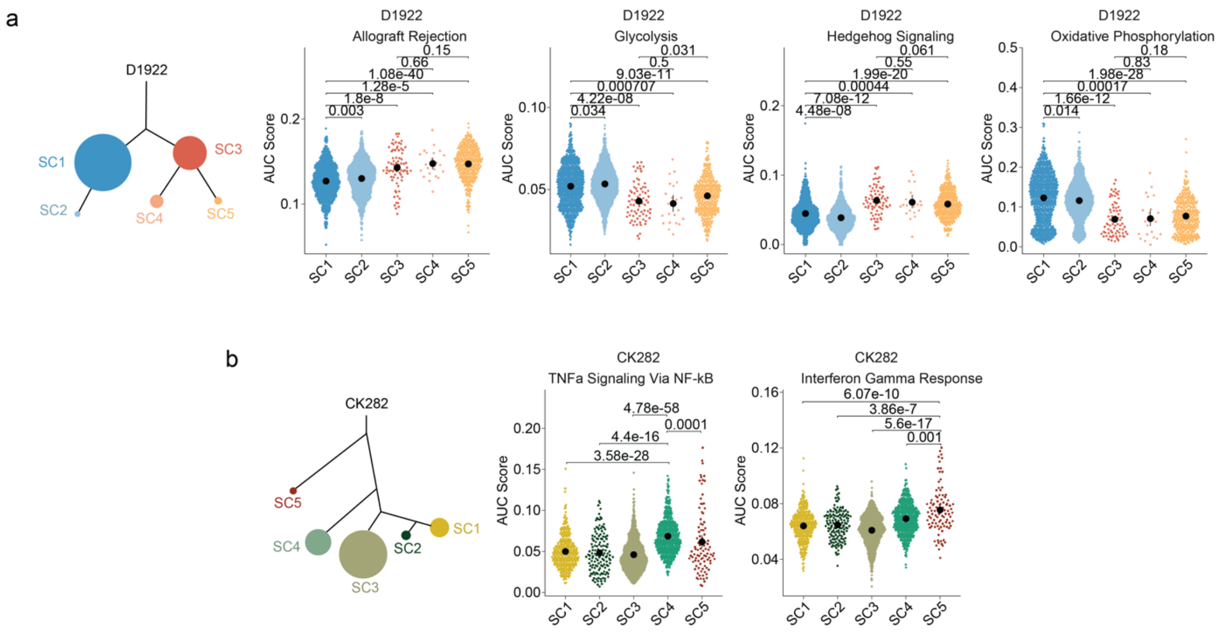

Supplementary Figure 7. Molecular expression networks in D1922 and CK282. **a** Area Under the Curve (AUC) score for the activity of the indicated gene sets for each cell in the different subclones in D1922 (n = 22 – 845 single cells). **b** Area Under the Curve score for the activity of the indicated gene sets for each cell in the different subclones in CK282 (n = 95 – 796 single cells). Cells are colored based on the subclones identified using scTRIP depicted in the clonal tree. Beeswarm plots show the 95% confidence interval for the mean and AUC scores were compared using two-tailed Wilcoxon test followed by Benjamini-Hochberg multiple correction testing.

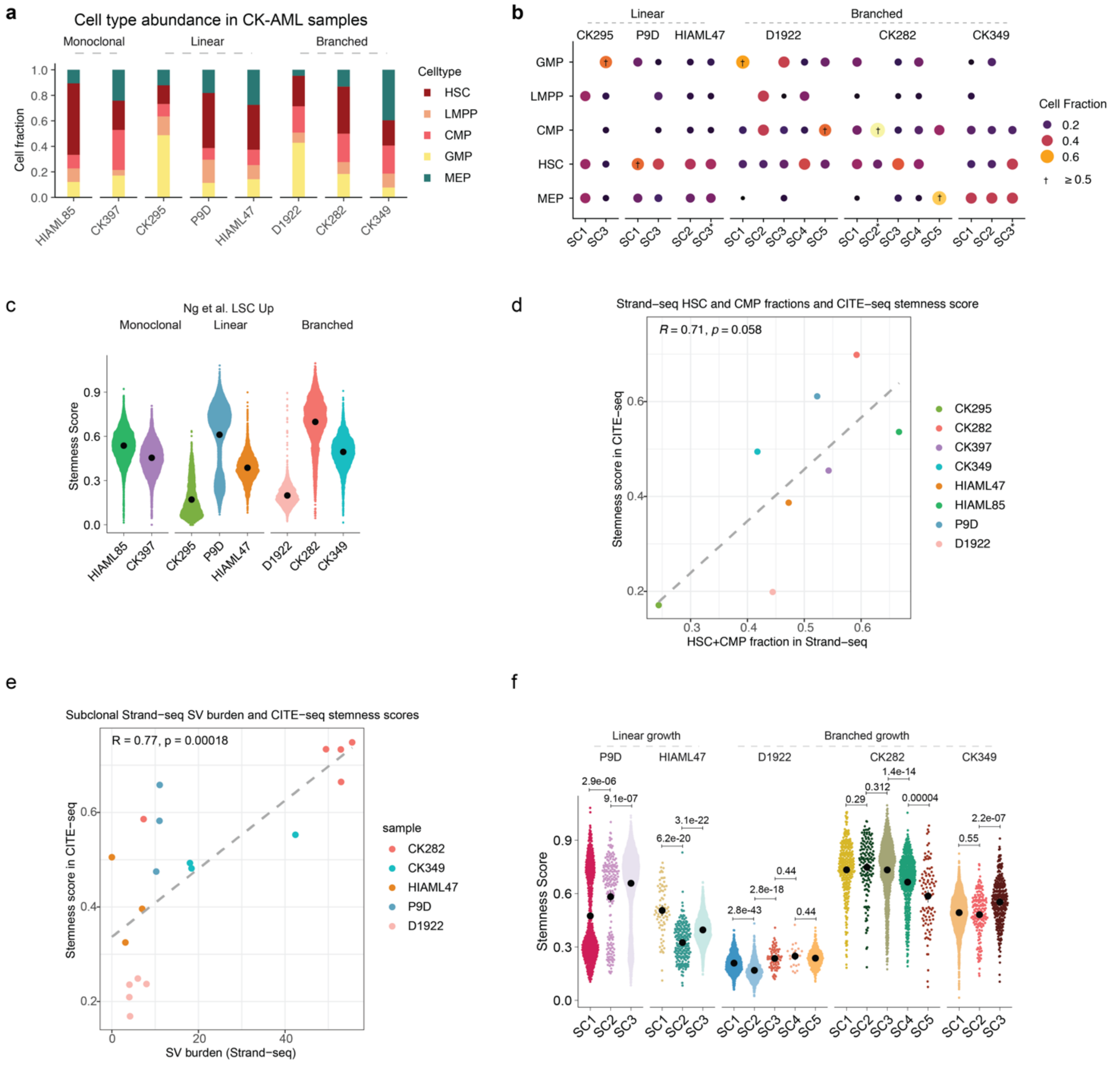

Supplementary Figure 8. Cell-type assignments and stemness features in CK-AML. a Stacked bar plots showing the fraction of indicated hematopoietic stem and progenitor cell-like states out of all cells in CK-AML patient samples. Cell types were annotated using a MNase-seq reference dataset from index sorted healthy CD34+ bone marrow cells, and cell typing was pursed using scNOVA. **b** Dot plots showing the fraction of indicated hematopoietic stem and progenitor cell-like states out of all cells in the different subclones from patients with linear and branched growth. Subclones consisting of ≥ 3 cells are shown. (*, Engraftment-driving subclone). **c** Expression of the Ng et al. LSC Up transcriptomic stemness score[12] in the single cells of the different primary samples at initial sampling. **d** Sum of HSC and CMP fractions in Strand-seq data vs. mean stemness scores in CITE-seq data. Each dot represents a sample and the dashed line shows the linear fit. Correlation was calculated using two-tailed Spearman correlation. **e** Mean subclonal structural variant (SV) burden in Strand-seq data vs. mean subclonal stemness scores in CITE-seq data. Each dot represents a subclone and the dashed line shows the linear fit. Correlation was calculated using two-tailed Spearman correlation. **f** Expression of the Ng et al. LSC Up transcriptomic stemness score[12] in the single cells of the different subclones. Stemness scores between subclones were compared using two-tailed Wilcoxon test followed by Benjamini-Hochberg multiple correction testing. Expression levels of the individual genes in the score were calculated from normalized and variance stabilized counts. In **c** and **f**, beeswarm plots show the 95% confidence interval for the mean. HSC: Hematopoietic stem cell, LMPP: Lympho-myeloid primed progenitor, CMP: Common myeloid progenitor, GMP: Granulocyte-macrophage progenitor, MEP: Megakaryocyte–erythroid progenitor.

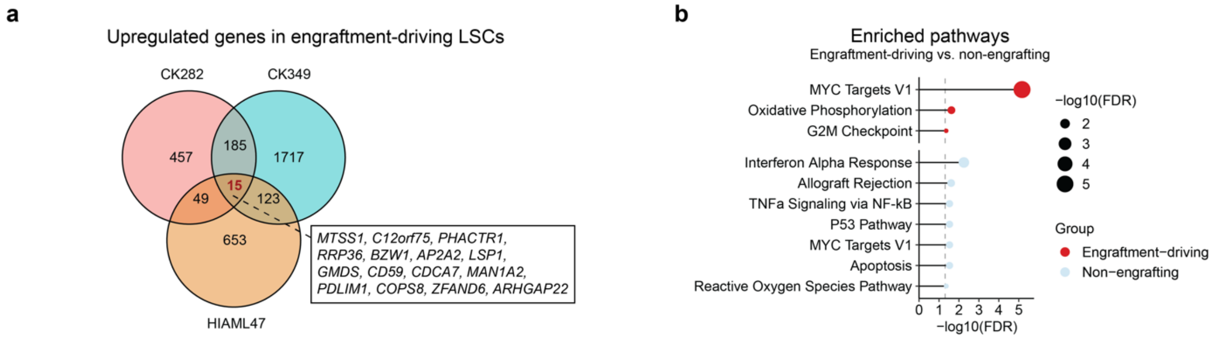

Supplementary Figure 9. Transcriptomic programs of engraftment-driving cells. a Genes upregulated in the engraftment-driving leukemic stem cells (LSCs) of CK282, CK349 and HIAML47 compared to non-engrafting cells. Genes with FDR < 0.05 and log-fold-change > 0.1 were included in the analysis. **b** Enriched pathways in engraftment-driving of LSCs of CK282, CK349 and HIAML47 compared to non-engrafting cells. Shared genes upregulated in two or more samples with FDR < 0.05 and log-fold-change > 0.1 were included in the analysis.

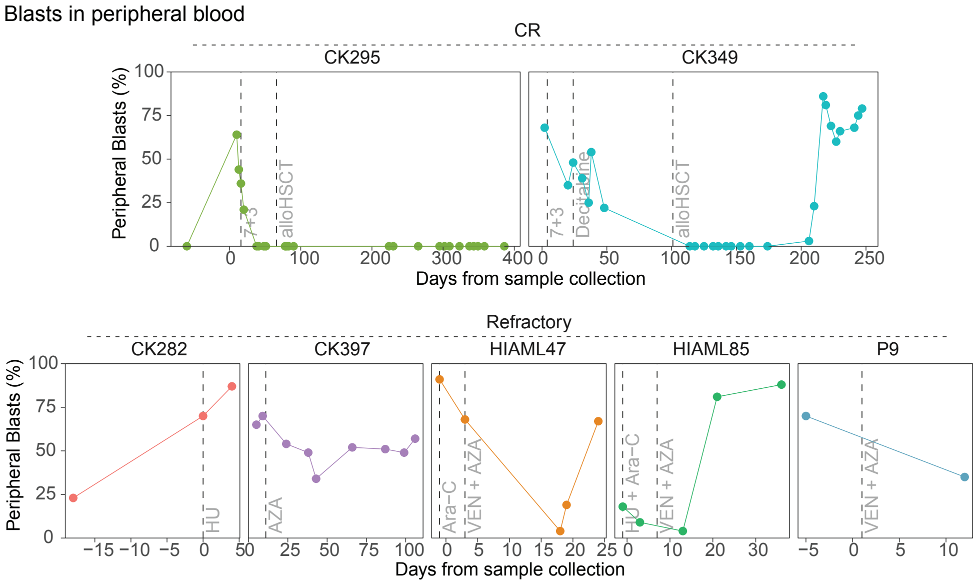

Supplementary Figure 10. Percentage of blasts in peripheral blood. Longitudinal peripheral blood counts for two patients achieving complete remission (CR) following treatment and five patients with refractory disease. Dashed lines indicate the start of the treatment with the treatment labelled next to the line. Day 0 represents the sample collection day from which Strand-seq and CITE-seq experiments were performed (see Fig. 1a). 7+3: Cytarabine + Daunorubicin, alloHSCT: allogeneic hematopoietic stem cell transplantation, HU: Hydroxyurea, AZA: Azacitidine, VEN: Venetoclax, Ara-C: Cytarabine.

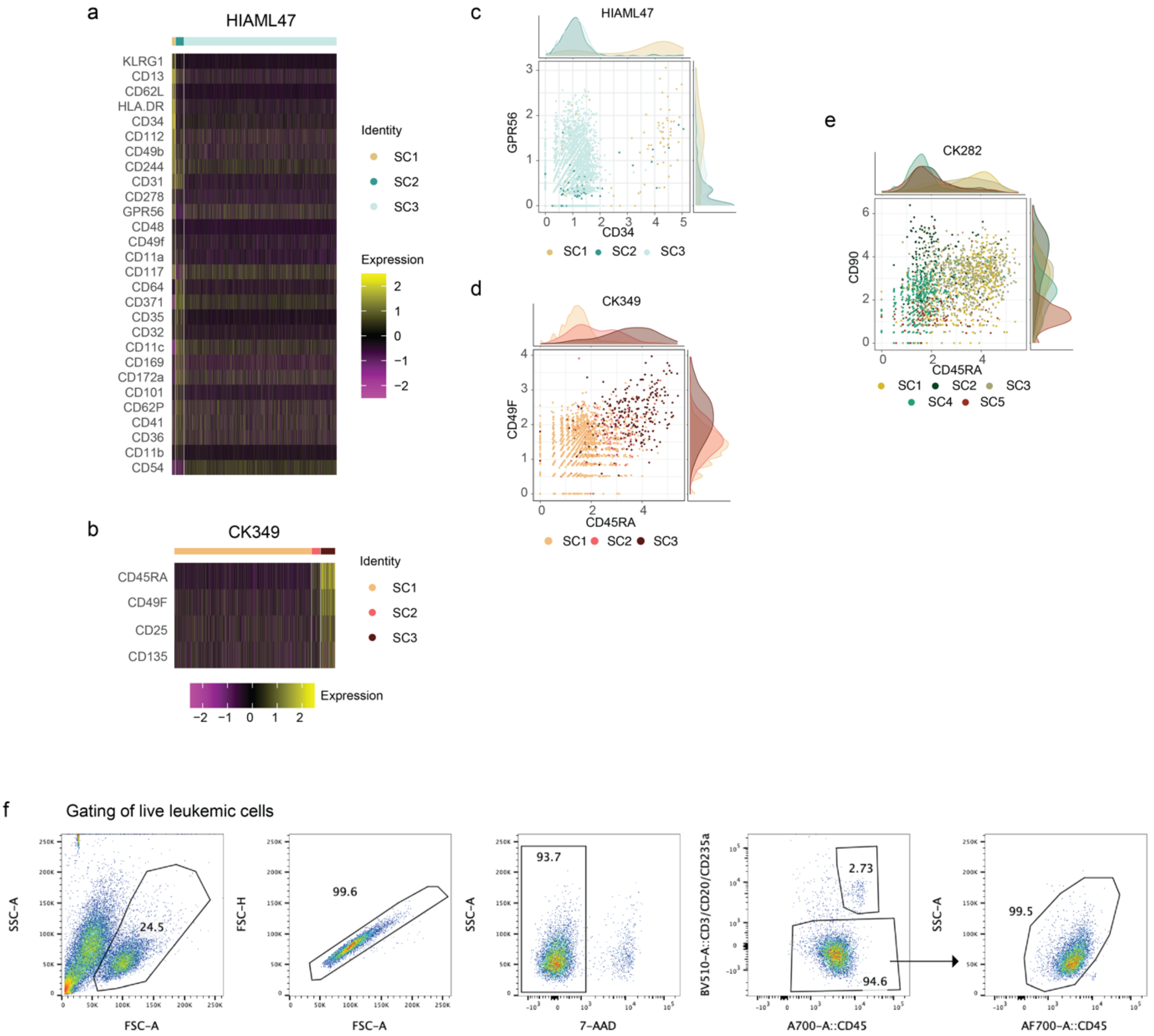

Supplementary Figure 11. Leveraging single-cell multi-omics to dissect drug response profiles. a-b Heatmap showing differentially expressed cell surface markers for each subclone in **a** HIAML47 and **b** CK349. **c** Scatter plot of CD34 and GPR56 cell surface expression as identified from HIAML47 CITE-seq data pre-gated to (pre-)leukemic cells. Shown are centered log ratio-transformed counts highlighted according to subclones. **d** Scatter plot of CD45RA and CD49F cell surface expression as identified from CK349 CITE-seq data pre-gated to leukemic cells. Shown are centered log ratio-transformed counts highlighted according to subclones. **e** Scatter plot of CD45RA and CD90 cell surface expression as identified from CK282 CITE-seq data pre-gated to leukemic cells. Shown are centered log ratio-transformed counts highlighted according to subclones. **f** Gating strategy for flow cytometry-based drug screening. Leukemic cells were gated as live lineage-negative (CD3-/CD20-/CD235a-) singlets within the CD45dim/SSClow/SSCmid gate (Fig. 5c, e-f, h-i, k-m, 7h-j, Extended Data Fig. 9a-g, 10h, Supplementary Fig. 12). The same strategy was used to sort leukemic cells for CITE-seq libraries with all CD45+ cells included. For intracellular staining of BCL-2 family members Zombie NIR was used instead of 7-AAD (Fig. 5n, Extended Data Fig. 9h).

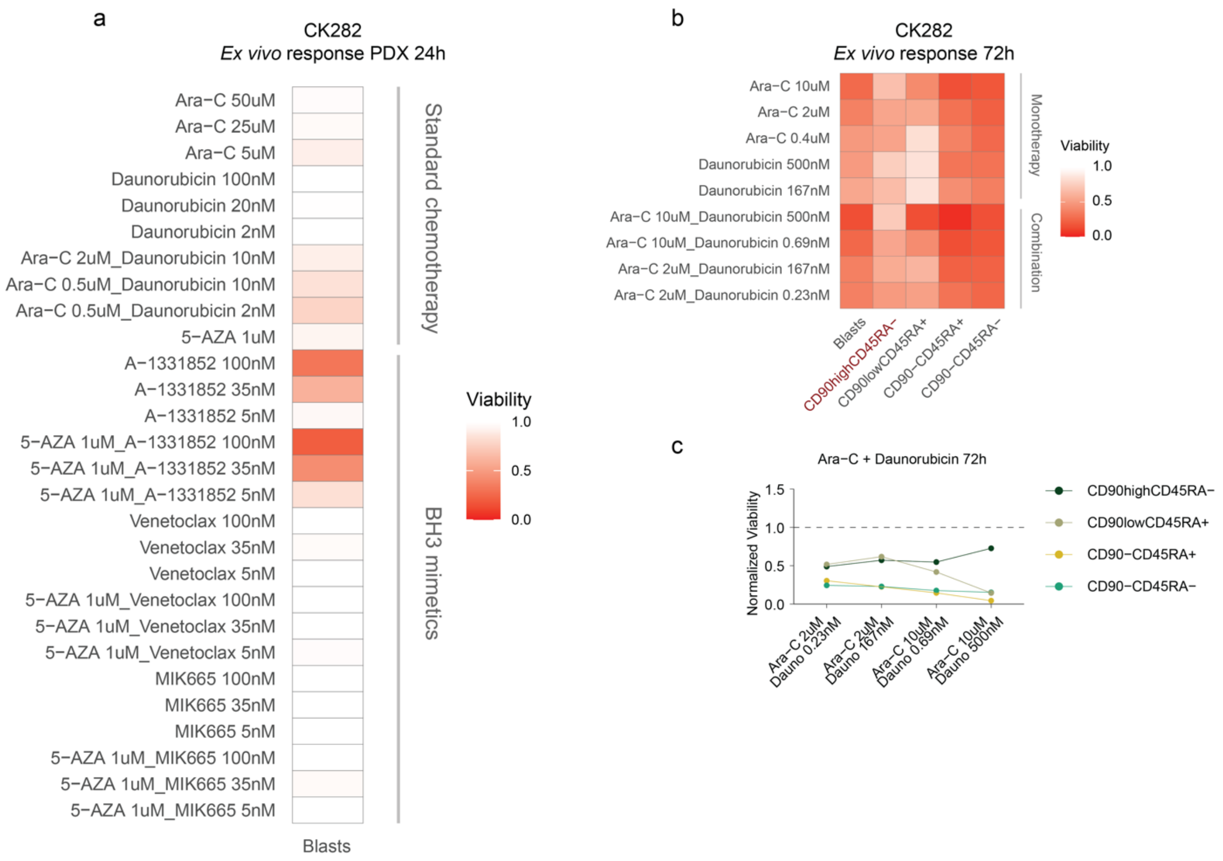

Supplementary Figure 12. *Ex vivo* drug response of CK282-derived cells. a Viabilities of human blasts after 24h *ex vivo* exposure with indicated concentrations of standard chemotherapy regimens as well as BH3 mimetics in the patient-derived xenograft (PDX) of CK282. Shown are the mean viabilities of two replicates. **b-c** Viabilities (fraction of viable cells compared to untreated control) of different populations after 72h *ex vivo* exposure with indicated concentrations of cytarabine and daunorubicin in CK282. In **b**, engraftment-driving population is highlighted in red.

Supplementary References

1. Stein, S. *et al.* Genomic instability and myelodysplasia with monosomy 7 consequent to EVI1 activation after gene therapy for chronic granulomatous disease. *Nat Med* **16**, 198-204 (2010).
2. Inaba, T., Honda, H. & Matsui, H. The enigma of monosomy 7. *Blood, The Journal of the American Society of Hematology* **131**, 2891-2898 (2018).
3. McClintock, B. The Stability of Broken Ends of Chromosomes in Zea Mays. *Genetics* **26**, 234-282 (1941).
4. Sanders, A.D. *et al.* Single-cell analysis of structural variations and complex rearrangements with tri-channel processing. *Nat Biotechnol* **38**, 343-354 (2020).
5. Rosswog, C. *et al.* Chromothripsis followed by circular recombination drives oncogene amplification in human cancer. *Nat Genet* **53**, 1673-1685 (2021).
6. Trumpp, A. & Haas, S. Cancer stem cells: The adventurous journey from hematopoietic to leukemic stem cells. *Cell* **185**, 1266-1270 (2022).
7. van Galen, P. *et al.* Single-Cell RNA-Seq Reveals AML Hierarchies Relevant to Disease Progression and Immunity. *Cell* **176**, 1265-1281 e24 (2019).
8. Grimes, K. *et al.* Cell-type-specific consequences of mosaic structural variants in hematopoietic stem and progenitor cells. *Nat Genet* **56**, 1134-1146 (2024).
9. Rodriguez-Meira, A. *et al.* Single-cell multi-omics identifies chronic inflammation as a driver of TP53-mutant leukemic evolution. *Nat Genet* **55**, 1531-1541 (2023).
10. Yamaoka, A. *et al.* EVI1 and GATA2 misexpression induced by inv(3)(q21q26) contribute to megakaryocyte-lineage skewing and leukemogenesis. *Blood Adv* **4**, 1722-1736 (2020).
11. Takeda, J. *et al.* Amplified EPOR/JAK2 Genes Define a Unique Subtype of Acute Erythroid Leukemia. *Blood Cancer Discov* **3**, 410-427 (2022).
12. Ng, S.W. *et al.* A 17-gene stemness score for rapid determination of risk in acute leukaemia. *Nature* **540**, 433-437 (2016).
13. Wojiski, S. *et al.* PML-RARalpha initiates leukemia by conferring properties of self-renewal to committed promyelocytic progenitors. *Leukemia* **23**, 1462-1471 (2009).
14. Rasmussen, M.L. *et al.* A Non-apoptotic Function of MCL-1 in Promoting Pluripotency and Modulating Mitochondrial Dynamics in Stem Cells. *Stem Cell Reports* **10**, 684-692 (2018).
15. Veas-Pérez de Tudela, M. *et al.* Regulation of Bcl-xL-ATP Synthase Interaction by Mitochondrial Cyclin B1-Cyclin-Dependent Kinase-1 Determines Neuronal Survival. *J Neurosci* **35**, 9287-9301 (2015).
16. Lucas, C.M. *et al.* High CIP2A levels correlate with an antiapoptotic phenotype that can be overcome by targeting BCL-XL in chronic myeloid leukemia. *Leukemia* **30**, 1273-1281 (2016).
17. Pollyea, D.A. *et al.* Venetoclax with azacitidine disrupts energy metabolism and targets leukemia stem cells in patients with acute myeloid leukemia. *Nat Med* **24**, 1859-1866 (2018).
18. Waclawiczek, A. *et al.* Combinatorial BCL2 Family Expression in Acute Myeloid Leukemia Stem Cells Predicts Clinical Response to Azacitidine/Venetoclax. *Cancer Discov* **13**, 1408-1427 (2023).
19. Zhang, Q. *et al.* Activation of RAS/MAPK pathway confers MCL-1 mediated acquired resistance to BCL-2 inhibitor venetoclax in acute myeloid leukemia. *Signal Transduct Target Ther* **7**, 51 (2022).
20. Griffiths, J.A., Scialdone, A. & Marioni, J.C. Using single-cell genomics to understand developmental processes and cell fate decisions. *Mol Syst Biol* **14**, e8046 (2018).
21. Sanders, A.D., Falconer, E., Hills, M., Spierings, D.C.J. & Lansdorp, P.M. Single-cell template strand sequencing by Strand-seq enables the characterization of individual homologs. *Nat Protoc* **12**, 1151-1176 (2017).
22. Jeong, H. *et al.* Functional analysis of structural variants in single cells using Strand-seq. *Nat Biotechnol* **41**, 832-844 (2023).
23. Griffith, M. *et al.* Optimizing cancer genome sequencing and analysis. *Cell Syst* **1**, 210-223 (2015).
24. Layer, R.M., Chiang, C., Quinlan, A.R. & Hall, I.M. LUMPY: a probabilistic framework for structural variant discovery. *Genome Biol* **15**, R84 (2014).
25. Davis, A., Gao, R. & Navin, N. Tumor evolution: Linear, branching, neutral or punctuated? *Biochim Biophys Acta Rev Cancer* **1867**, 151-161 (2017).

26. Bochtler, T. *et al.* Clonal heterogeneity as detected by metaphase karyotyping is an indicator of poor prognosis in acute myeloid leukemia. *J Clin Oncol* **31**, 3898-3905 (2013).
27. Burrell, R.A., McGranahan, N., Bartek, J. & Swanton, C. The causes and consequences of genetic heterogeneity in cancer evolution. *Nature* **501**, 338-345 (2013).
28. Hu, Z., Li, Z., Ma, Z. & Curtis, C. Multi-cancer analysis of clonality and the timing of systemic spread in paired primary tumors and metastases. *Nat Genet* **52**, 701-708 (2020).
29. Al Bakir, M. *et al.* The evolution of non-small cell lung cancer metastases in TRACERx. *Nature* **616**, 534-542 (2023).
30. Morita, K. *et al.* Clonal evolution of acute myeloid leukemia revealed by high-throughput single-cell genomics. *Nat Commun* **11**, 5327 (2020).
31. Rausch, T. *et al.* Genome sequencing of pediatric medulloblastoma links catastrophic DNA rearrangements with TP53 mutations. *Cell* **148**, 59-71 (2012).
32. Cosenza, M.R., Rodriguez-Martin, B. & Korbel, J.O. Structural Variation in Cancer: Role, Prevalence, and Mechanisms. *Annu Rev Genomics Hum Genet* **23**, 123-152 (2022).
33. Garsed, D.W. *et al.* The architecture and evolution of cancer neochromosomes. *Cancer Cell* **26**, 653-667 (2014).
34. Sansregret, L., Vanhaesebroeck, B. & Swanton, C. Determinants and clinical implications of chromosomal instability in cancer. *Nat Rev Clin Oncol* **15**, 139-150 (2018).
