# Supplementary material for: Single-cell multiomics analysis reveals dynamic clonal evolution and targetable phenotypes in acute myeloid leukemia with complex karyotype
Source: Nat Genet. 2024 Nov 25;56(12):2790–803. doi: 10.1038/s41588-024-01999-x (PMC11631769; doi:10.1038/s41588-024-01999-x)
Supplement: Supplementary file 2 — Reporting Summary [file 41588_2024_1999_MOESM2_ESM.pdf]

nature portfolio

Corresponding author(s): Andreas Trumpp
Jan Korbel

Last updated by author(s): 20.09.2024

Reporting Summary

Nature Portfolio wishes to improve the reproducibility of the work that we publish. This form provides structure for consistency and transparency in reporting. For further information on Nature Portfolio policies, see our Editorial Policies and the Editorial Policy Checklist.

Statistics

For all statistical analyses, confirm that the following items are present in the figure legend, table legend, main text, or Methods section.

n/a	Confirmed	
☐	☒	The exact sample size (*n*) for each experimental group/condition, given as a discrete number and unit of measurement
☐	☒	A statement on whether measurements were taken from distinct samples or whether the same sample was measured repeatedly
☐	☒	The statistical test(s) used AND whether they are one- or two-sided<br>*Only common tests should be described solely by name; describe more complex techniques in the Methods section.*
☒	☐	A description of all covariates tested
☐	☒	A description of any assumptions or corrections, such as tests of normality and adjustment for multiple comparisons
☐	☒	A full description of the statistical parameters including central tendency (e.g. means) or other basic estimates (e.g. regression coefficient) AND variation (e.g. standard deviation) or associated estimates of uncertainty (e.g. confidence intervals)
☐	☒	For null hypothesis testing, the test statistic (e.g. *F*, *t*, *r*) with confidence intervals, effect sizes, degrees of freedom and *P* value noted<br>*Give P values as exact values whenever suitable.*
☒	☐	For Bayesian analysis, information on the choice of priors and Markov chain Monte Carlo settings
☒	☐	For hierarchical and complex designs, identification of the appropriate level for tests and full reporting of outcomes
☐	☒	Estimates of effect sizes (e.g. Cohen's *d*, Pearson's *r*), indicating how they were calculated

Our web collection on statistics for biologists contains articles on many of the points above.

Software and code

Policy information about availability of computer code

Data collection	Our study uses data output by illumina sequencers and bionano saphyr optical genome mapping system and hence no special software was used for collecting it.
Data analysis	The computational software used in the study include scNOVA (https://github.com/jeongdo801/scNOVA), Mosaicatcher (https://github.com/friendsofstrandseq/mosaicatcher-pipeline), Strand-PhaseR (https://github.com/daewoooo/StrandPhaseR), CONICSmat (https://github.com/diazlab/CONICS), Delly2 (https://github.com/dellytools/delly), NO_based_HSPC_classifier (https://github.com/jeongdo801/NO_based_HSPC_classifier), PloidyAssignR (https://github.com/lysfyg/PloidyAssignR), BWA (v.0.7.15), STAR (v.2.7.9a and v.2.5.3a), SAMtools (v.1.3.1), biobambam2 (v.2.0.76), Sambamba (v.0.6.5), R (v.4.0.0), DESeq2, Cell Ranger (v.6.0), Seurat (v.4.3.0.1), scran (1.28.2), AUCell (v.1.2.2.0), SingleR (2.2.0), Arriba (v.1.2.0), FlowJo (v.10.5.3), Prism (v.9.3.1), Bionano Solve software (v3.7), Bionano Access Software (v1.7.1), and BD FACSDiva. Analysis notebooks for the figures are available at: https://github.com/amleppa/scNOVA-CITE_paper.

For manuscripts utilizing custom algorithms or software that are central to the research but not yet described in published literature, software must be made available to editors and reviewers. We strongly encourage code deposition in a community repository (e.g. GitHub). See the Nature Portfolio guidelines for submitting code & software for further information.

Data

Policy information about availability of data

All manuscripts must include a data availability statement. This statement should provide the following information, where applicable:

- Accession codes, unique identifiers, or web links for publicly available datasets
- A description of any restrictions on data availability
- For clinical datasets or third party data, please ensure that the statement adheres to our policy

Sequencing data from this study can be retrieved from the European Genome-phenome Archive (EGA) and ArrayExpress. Data from primary CK-AML cells and PDXs are available under the following accession numbers: Strand-seq and CITE-seq (EGAS00001007436); bulk RNA-seq (E-MTAB-14420). Access to human patient data deposited at EGA is governed by the EGA Data Access Committee. We also used publicly available databases as follows: human GRCh38 reference database (ENSEMBL; http://ftp.ensembl.org/) and Molecular signature database (MSigDB; https://www.gsea-msigdb.org/gsea/msigdb/).

Research involving human participants, their data, or biological material

Policy information about studies with human participants or human data. See also policy information about sex, gender (identity/presentation), and sexual orientation and race, ethnicity and racism.

Reporting on sex and gender	Patient sex was reported in accordance with the sex identified on the national identification card. We analyzed 8 CK-AML samples, four with female sex and four with male sex. Sex was not considered in the study design since this study focuses on intra-sample comparison in a case-based manner rather than on performing statistical tests between groups of samples.
Reporting on race, ethnicity, or other socially relevant groupings	We analyzed samples from 8 CK-AML patients (samples obtained at diagnosis/salvage stage and relapse/refractory stage). Clinical covariates of CK-AML patients are given in Supplementary Table 1.
Population characteristics	We analyzed samples from 8 CK-AML patients (samples obtained at diagnosis/salvage stage and relapse/refractory stage). Clinical covariates of CK-AML patients are given in Supplementary Table 1.
Recruitment	All samples were obtained from patients that provided written informed consent for the research use of their specimens in agreement with the Declaration of Helsinki. The project was approved by the Ethics Committee/Institutional Review Board of the Medical Faculty of Heidelberg and Cancer and Leukemia Group B (GALGB) (NCT-MASTER Platform S-206/2011 and S-169/2017, and GALGB studies CALGB 8461, CALGB 9665 and CALGB 20202). The protocols involved collection of bone marrow (BM) aspirates and peripheral blood (PB) samples. Part of the cohort were provided by the NCT cell and liquid biobank, a member of the BMBH. Samples included in the study were chosen based on their clinical classification as complex karyotype and the availability of at least two viably-frozen sample vials.
Ethics oversight	The project was approved by the Ethics Committee/Institutional Review Board of the Medical Faculty of Heidelberg and Cancer and Leukemia Group B (GALGB) (NCT-MASTER Platform S-206/2011 and S-169/2017, and GALGB studies CALGB 8461, CALGB 9665 and CALGB 20202).

Note that full information on the approval of the study protocol must also be provided in the manuscript.

Field-specific reporting

Please select the one below that is the best fit for your research. If you are not sure, read the appropriate sections before making your selection.

☒ Life sciences ☐ Behavioural & social sciences ☐ Ecological, evolutionary & environmental sciences

For a reference copy of the document with all sections, see nature.com/documents/nr-reporting-summary-flat.pdf

Life sciences study design

All studies must disclose on these points even when the disclosure is negative.

Sample size	No sample-size calculation was performed since this study focuses on intra-sample comparison in a case-based manner rather than on performing statistical tests between groups of samples. The cohort size was determined by the number of CK-AML samples available.
Data exclusions	In Strand-seq data, we excluded low quality single-cell libraries that showed very low, uneven coverage, of an excess of 'background reads' yielding noisy single cell data prior to analysis. Cells with incomplete BrdU incorporation or cells undergoing more than one DNA synthesis phase under BrdU exposure are largely excluded during cell sorting and thus get only rarely sequenced during Strand-seq experiments.<br>In CITE-seq data, we excluded cells from the analysis if fewer than 200 or more than 8,000 distinct genes, fewer than 1,000 counts or more than 15% of reads mapping to mitochondrial genes were detected.
Replication	We have repeated the analyses of our datasets a minimum of two times and can confirm consistent and reproducible results from the workflows used. To ensure reproducibility of our experimental findings, we generated replicates wherever possible confirming the reproducibility of the results. Use of replicates is indicated in the figure legends. All attempts of replication were successful.

Randomization	Does not apply (there are no experimental groups in our study).
Blinding	Does not apply (the study focuses on intra-sample comparison rather than performing statistical tests between groups of samples).

Reporting for specific materials, systems and methods

We require information from authors about some types of materials, experimental systems and methods used in many studies. Here, indicate whether each material, system or method listed is relevant to your study. If you are not sure if a list item applies to your research, read the appropriate section before selecting a response.

Materials & experimental systems

n/a	Involved in the study	
☐	☒	Antibodies
☒	☐	Eukaryotic cell lines
☒	☐	Palaeontology and archaeology
☐	☒	Animals and other organisms
☒	☐	Clinical data
☒	☐	Dual use research of concern
☒	☐	Plants

Methods

n/a	Involved in the study	
☒	☐	ChIP-seq
☐	☒	Flow cytometry
☒	☐	MRI-based neuroimaging

Antibodies

Antibodies used

FACS (clone; manufacturer; catalogue number; application; reactivity):
FITC mouse anti-human mCD45 (clone 30-F11; eBioscience; # 11-0451-82; FC; Mouse)
AF700 mouse anti-human mCD45 (clone 30-F11; BioLegend; # 103128; FC; Mouse)
BV421 mouse anti-human CD45RA (clone HI100; BD Biosciences; # 562885; FC; Human)
BV510 mouse anti-human CD3 (clone UCHT1; BioLegend; # 317332; FC; Human)
BV510 mouse anti-human CD20 (clone 2H7; BioLegend; # 302340; FC; Human)
BV510 mouse anti-human CD235a (clone GAR2; BD Biosciences; # 740174; FC; Human)
AF700 mouse anti-human CD45 (clone HI30; BD Biosciences; # 560566; FC; Human)
FITC mouse anti-human CD45 (clone HI30; BioLegend; # 304006; FC; Human)
APC-Cy7 mouse anti-human CD45 (clone HI30; BioLegend; # 304014; FC; Human)
PE mouse anti-human CD49F (clone GoH3; BioLegend; # 313611; FC; Human, Mouse, Cynomolgus, Rhesus)
PE-Cy7 mouse anti-human GPR56 (clone CG4; BioLegend; # 358206; FC; Human, Mouse)
BUV395 mouse anti-human CD34 (clone 581; BD Biosciences; # 563778; FC; Human)
BUV496 mouse anti-human CD38 (clone HIT2; BD Biosciences; # 612946; FC; Human)
FITC mouse anti-human CD99 (clone HCD99; BioLegend; # 318006; FC; Human)
PE mouse anti-human CD90 (clone 5E10; BioLegend; # 328109; FC; Human)
BV711 mouse anti-human CD13 (clone WM15; BioLegend; # 301721; FC; Human)
BV785 mouse anti-human CD117 (clone 104D2; BioLegend; # 313238; FC; Human)
PE mouse anti-human HLA-DR (clone L243; BioLegend; # 307605; FC; Human, Cynomolgus, Rhesus)
PE-CF594 mouse anti-human CD123 (clone 7G3; BD Biosciences; # 562391; FC; Human)
FITC mouse anti-human CD81 (clone 5A6; BioLegend; # 349504; FC; Human)
APC mouse anti-human CD18 (clone TS1/18; BioLegend; # 302114; FC; Human)
AF700 mouse anti-human CD54 (clone HA58; BioLegend; # 353126; FC; Human)
BV711 mouse anti-human CD11b (clone ICRF44; BioLegend; # 301344; FC; Human, Cynomolgus, Rhesus)
BV786 mouse anti-human CD33 (clone WM53; BD Biosciences; # 740974; FC; Human)
FITC mouse anti-human CD9 (clone HI9a ; BioLegend; # 312104; FC; Human)
APC mouse anti-human CD64 (clone 10.1 ; BioLegend; # 305014; FC; Human, Cynomolgus, Rhesus)
BV711 mouse anti-human CD45 (clone HI30; BD Biosciences; # 564357; FC; Human)
PE mouse anti-human CD11b (clone ICRF44; BioLegend; # 982606; FC; Human)
AF488 mouse anti-human MCL-1 (clone D2W9E; Cell Signaling; # 58326; FC; Human, Mouse, Rat)
AF647 mouse anti-human BCL-2 (clone 124; Cell Signaling; # 82655; FC; Human)
PE-Cy7 mouse anti-human BCL-xL (clone 54H6; Cell Signaling; # 81965; FC; Human, Mouse, Rat, Monkey)
Zombie NIR (clone -; BioLegend; # 423105; FC; All species)
7-AAD (clone -; BD Biosciences; # 559925; FC; All species)

CITE-seq (clone; manufacturer; catalogue number; application; reactivity):
Total-seq A mouse anti-human CD26 (clone BA5b; BioLegend; # 302720; PG; Human)
Total-seq A mouse anti-human CD45 (clone HI30; BioLegend; # 304064; PG; Human)
Total-seq A mouse anti-human TIM3 (clone F38-2E2; BioLegend; # 345047; PG; Human)
Total-seq A mouse anti-human CD99 (clone 3B2/TA8; BioLegend; # 371317; PG; Human)
Total-seq A mouse anti-human CD33 (clone P67.6; BioLegend; # 366629; PG; Human)
Total-seq A mouse anti-human CD38 (clone HIT2; BioLegend; # 303541; PG; Human)
Total-seq A mouse anti-human CD44 (clone IM7; BioLegend; # 103045; PG; Human, Mouse)
Total-seq A mouse anti-human CD117 (clone 104D2; BioLegend; # 313241; PG; Human)
Total-seq A mouse anti-human CD34 (clone 581; BioLegend; # 343537; PG; Human)
Total-seq A mouse anti-human CD90 (clone 5E10; BioLegend; # 328135; PG; Human)

Total-seq A mouse anti-human CD49F (clone GoH3; BioLegend; # 313633; PG; Human, Mouse, Cynomolgus, Rhesus)
Total-seq A mouse anti-human CD10 (clone HI10a; BioLegend; # 312231; PG; Human, Cynomolgus, Rhesus)
Total-seq A mouse anti-human CD135 (clone BV10AH2; BioLegend; # 313317; PG; Human)
Total-seq A mouse anti-human CD123 (clone 6H6; BioLegend; # 306037; PG; Human)
Total-seq A mouse anti-human CD371 (CLEC12A) (clone 50C1; BioLegend; # 353613; PG; Human)
Total-seq A mouse anti-human CD7 (clone CD7-6B7; BioLegend; # 343123; PG; Human)
Total-seq A mouse anti-human HLA-DR (clone L243; BioLegend; # 307659; PG; Human, Cynomolgus, Rhesus)
Total-seq A mouse anti-human GPR56 (clone CG4; BioLegend; # 358207; PG; Human, Mouse)
Total-seq A mouse anti-human CD45RA (clone HI100; BioLegend; # 304157; PG; Human)
Total-seq A mouse anti-human CD64 (clone 10.1; BioLegend; # 305037; PG; Human, Cynomolgus, Rhesus)
Total-seq A mouse anti-human CD11b (clone ICRF44; BioLegend; # 301353; PG; Human, Cynomolgus, Rhesus)
Total-seq A mouse anti-human CD3 (clone UCHT1; BioLegend; # 300475; PG; Human)
Total-seq A mouse anti-human CD4 (clone SK3; BioLegend; # 344649; PG; Human)
Total-seq A mouse anti-human CD8 (clone SK1; BioLegend; # 344751; PG; Human, Cynomolgus, Rhesus)
Total-seq A mouse anti-human CD25 (clone BC96; BioLegend; # 302643; PG; Human)
Total-seq A mouse anti-human CD19 (clone HIB19; BioLegend; # 302259; PG; Human)
Total-seq A mouse anti-human CD56 (clone 5.1H11; BioLegend; # 362557; PG; Human)
Total-seq A mouse anti-human CD16 (clone 3G8; BioLegend; # 302061; PG; Human, Cynomolgus, Rhesus)
Total-seq A mouse anti-human CD274 (PD-L1) (clone 29E.2A3; BioLegend; # 329743; PG; Human)
Total-seq A mouse anti-human CD223 (LAG-3) (clone 11C3C65; BioLegend; # 369333; PG; Human)
Total-seq A mouse anti-human CD152 (CTLA-4) (clone BNI3; BioLegend; # 369619; PG; Human)
Total-seq A mouse anti-human CD279 (PD-1) (clone EH12.2H7; BioLegend; # 329955; PG; Human)
Total-seq A mouse anti-human CD86 (clone IT2.2; BioLegend; # 305443; PG; Human, Cynomolgus, Rhesus)
Total-seq A mouse anti-human CD226 (DNAM-1) (clone 11A8; BioLegend; # 338335; PG; Human)
Total-seq A mouse anti-human CD314 (NKG2D) (clone 1D11; BioLegend; # 320835; PG; Human)
Total-seq A mouse anti-human CD119 (IFNGR1) (clone GIR-208; BioLegend; # 308607; PG; Human)
Total-seq A mouse anti-human CD155 (PVR) (clone SKII.4; BioLegend; # 337623; PG; Human)
Total-seq A mouse anti-human Streptavidin (clone -; BioLegend; # 405251; PG; All Species)
Total-seq A mouse anti-human pan-NK2GDL (clone -; R&D Systems; # 1299-NK-050; Binding Activity; Human)
Total-seq B mouse anti-human CD86 (clone IT2.2; BioLegend; # 399904; PG; Human)
Total-seq B mouse anti-human CD274 (B7-H1, PD-L1) (clone 29E.2A3; BioLegend; # 399904; PG; Human)
Total-seq B mouse anti-human CD270 (HVEM, TR2) (clone 122; BioLegend; # 399904; PG; Human)
Total-seq B mouse anti-human CD155 (PVR) (clone SKII.4; BioLegend; # 399904; PG; Human)
Total-seq B mouse anti-human CD112 (Nectin-2) (clone TX31; BioLegend; # 399904; PG; Human)
Total-seq B mouse anti-human CD47 (clone CC2C6; BioLegend; # 399904; PG; Human)
Total-seq B mouse anti-human CD48 (clone BJ40; BioLegend; # 399904; PG; Human)
Total-seq B mouse anti-human CD40 (clone 5C3; BioLegend; # 399904; PG; Human)
Total-seq B mouse anti-human CD154 (clone 24-31; BioLegend; # 399904; PG; Human)
Total-seq B mouse anti-human CD52 (clone HI186; BioLegend; # 399904; PG; Human)
Total-seq B mouse anti-human CD3 (clone UCHT1; BioLegend; # 399904; PG; Human)
Total-seq B mouse anti-human CD8 (clone SK1; BioLegend; # 399904; PG; Human)
Total-seq B mouse anti-human CD56 (NCAM) (clone 5.1H11; BioLegend; # 399904; PG; Human)
Total-seq B mouse anti-human CD19 (clone HIB19; BioLegend; # 399904; PG; Human)
Total-seq B mouse anti-human CD33 (clone P67.6; BioLegend; # 399904; PG; Human)
Total-seq B mouse anti-human CD11c (clone S-HCL-3; BioLegend; # 399904; PG; Human)
Total-seq B mouse anti-human HLA-A,B,C (clone W6/32; BioLegend; # 399904; PG; Human)
Total-seq B mouse anti-human CD45RA (clone HI100; BioLegend; # 399904; PG; Human)
Total-seq B mouse anti-human CD123 (clone 6H6; BioLegend; # 399904; PG; Human)
Total-seq B mouse anti-human CD7 (clone CD7-6B7; BioLegend; # 399904; PG; Human)
Total-seq B mouse anti-human CD105 (clone 43A3; BioLegend; # 399904; PG; Human)
Total-seq B mouse anti-human anti-human/mouse CD49f (clone GoH3; BioLegend; # 399904; PG; Human)
Total-seq B mouse anti-human CD194 (CCR4) (clone L291H4; BioLegend; # 399904; PG; Human)
Total-seq B mouse anti-human CD4 (clone RPA-T4; BioLegend; # 399904; PG; Human)
Total-seq B mouse anti-human anti-mouse/human CD44 (clone IM7; BioLegend; # 399904; PG; Human)
Total-seq B mouse anti-human CD14 (clone M5E2; BioLegend; # 399904; PG; Human)
Total-seq B mouse anti-human CD16 (clone 3G8; BioLegend; # 399904; PG; Human)
Total-seq B mouse anti-human CD25 (clone BC96; BioLegend; # 399904; PG; Human)
Total-seq B mouse anti-human CD45RO (clone UCHL1; BioLegend; # 399904; PG; Human)
Total-seq B mouse anti-human CD279 (PD-1) (clone EH12.2H7; BioLegend; # 399904; PG; Human)
Total-seq B mouse anti-human TIGIT (VSTM3) (clone A15153G; BioLegend; # 399904; PG; Human)
Total-seq B mouse anti-human Mouse IgG1, κ isotype (clone MOPC-21; BioLegend; # 399904; PG; Human)
Total-seq B mouse anti-human Mouse IgG2a, κ isotype (clone MOPC-173; BioLegend; # 399904; PG; Human)
Total-seq B mouse anti-human Mouse IgG2b, κ isotype (clone MPC-11; BioLegend; # 399904; PG; Human)
Total-seq B mouse anti-human Rat IgG2b, κ isotype (clone RTK4530; BioLegend; # 399904; PG; Human)
Total-seq B mouse anti-human CD20 (clone 2H7; BioLegend; # 399904; PG; Human)
Total-seq B mouse anti-human CD335 (NKp46) (clone 9E2; BioLegend; # 399904; PG; Human)
Total-seq B mouse anti-human CD31 (clone WM59; BioLegend; # 399904; PG; Human)
Total-seq B mouse anti-human Podoplanin (clone NC-08; BioLegend; # 399904; PG; Human)
Total-seq B mouse anti-human CD146 (clone P1H12; BioLegend; # 399904; PG; Human)
Total-seq B mouse anti-human IgM (clone MHM-88; BioLegend; # 399904; PG; Human)
Total-seq B mouse anti-human CD5 (clone UCHT2; BioLegend; # 399904; PG; Human)
Total-seq B mouse anti-human CD195 (CCR5) (clone J418F1; BioLegend; # 399904; PG; Human)
Total-seq B mouse anti-human CD32 (clone FUN-2; BioLegend; # 399904; PG; Human)
Total-seq B mouse anti-human CD196 (CCR6) (clone G034E3; BioLegend; # 399904; PG; Human)
Total-seq B mouse anti-human CD185 (CXCR5) (clone J252D4; BioLegend; # 399904; PG; Human)
Total-seq B mouse anti-human CD103 (Integrin αE) (clone Ber-ACT8; BioLegend; # 399904; PG; Human)

Total-seq B mouse anti-human CD69 (clone FN50; BioLegend; # 399904; PG; Human)
Total-seq B mouse anti-human CD62L (clone DREG-56; BioLegend; # 399904; PG; Human)
Total-seq B mouse anti-human CD161 (clone HP-3G10; BioLegend; # 399904; PG; Human)
Total-seq B mouse anti-human CD152 (CTLA-4) (clone BNI3; BioLegend; # 399904; PG; Human)
Total-seq B mouse anti-human CD223 (LAG-3) (clone 11C3C65; BioLegend; # 399904; PG; Human)
Total-seq B mouse anti-human KLRG1 (MAFA) (clone SA231A2; BioLegend; # 399904; PG; Human)
Total-seq B mouse anti-human CD27 (clone O323; BioLegend; # 399904; PG; Human)
Total-seq B mouse anti-human CD107a (LAMP-1) (clone H4A3; BioLegend; # 399904; PG; Human)
Total-seq B mouse anti-human CD95 (Fas) (clone DX2; BioLegend; # 399904; PG; Human)
Total-seq B mouse anti-human CD134 (OX40) (clone Ber-ACT35 (ACT35); BioLegend; # 399904; PG; Human)
Total-seq B mouse anti-human HLA-DR (clone L243; BioLegend; # 399904; PG; Human)
Total-seq B mouse anti-human CD1c (clone L161; BioLegend; # 399904; PG; Human)
Total-seq B mouse anti-human CD11b (clone ICRF44; BioLegend; # 399904; PG; Human)
Total-seq B mouse anti-human CD64 (clone 10.1; BioLegend; # 399904; PG; Human)
Total-seq B mouse anti-human CD141 (Thrombomodulin) (clone M80; BioLegend; # 399904; PG; Human)
Total-seq B mouse anti-human CD1d (clone 51.1; BioLegend; # 399904; PG; Human)
Total-seq B mouse anti-human CD314 (NKG2D) (clone 1D11; BioLegend; # 399904; PG; Human)
Total-seq B mouse anti-human CD35 (clone E11; BioLegend; # 399904; PG; Human)
Total-seq B mouse anti-human CD57 Recombinant (clone QA17A04; BioLegend; # 399904; PG; Human)
Total-seq B mouse anti-human CD272 (BTLA) (clone MIH26; BioLegend; # 399904; PG; Human)
Total-seq B mouse anti-human anti-human/mouse/rat CD278 (ICOS) (clone C398.4A; BioLegend; # 399904; PG; Human)
Total-seq B mouse anti-human CD58 (LFA-3) (clone TS2/9; BioLegend; # 399904; PG; Human)
Total-seq B mouse anti-human CD39 (clone A1; BioLegend; # 399904; PG; Human)
Total-seq B mouse anti-human CX3CR1 (clone K0124E1; BioLegend; # 399904; PG; Human)
Total-seq B mouse anti-human CD24 (clone ML5; BioLegend; # 399904; PG; Human)
Total-seq B mouse anti-human CD21 (clone Bu32; BioLegend; # 399904; PG; Human)
Total-seq B mouse anti-human CD11a (clone TS2/4; BioLegend; # 399904; PG; Human)
Total-seq B mouse anti-human CD79b (Igβ) (clone CB3-1; BioLegend; # 399904; PG; Human)
Total-seq B mouse anti-human CD244 (2B4) (clone C1.7; BioLegend; # 399904; PG; Human)
Total-seq B mouse anti-human CD169 (clone 7-239; BioLegend; # 399904; PG; Human)
Total-seq B mouse anti-human anti-human/mouse integrin β7 (clone FIB504; BioLegend; # 399904; PG; Human)
Total-seq B mouse anti-human CD268 (BAFF-R) (clone 11C1; BioLegend; # 399904; PG; Human)
Total-seq B mouse anti-human CD42b (clone HIP1; BioLegend; # 399904; PG; Human)
Total-seq B mouse anti-human CD54 (clone HA58; BioLegend; # 399904; PG; Human)
Total-seq B mouse anti-human CD62P (P-Selectin) (clone AK4; BioLegend; # 399904; PG; Human)
Total-seq B mouse anti-human CD119 (IFN-γ R α chain) (clone GIR-208; BioLegend; # 399904; PG; Human)
Total-seq B mouse anti-human TCR α/β (clone IP26; BioLegend; # 399904; PG; Human)
Total-seq B mouse anti-human Rat IgG1, κ isotype (clone RTK2071; BioLegend; # 399904; PG; Human)
Total-seq B mouse anti-human Rat IgG2a, κ Isotype (clone RTK2758; BioLegend; # 399904; PG; Human)
Total-seq B mouse anti-human CD192 (CCR2) (clone K036C2; BioLegend; # 399904; PG; Human)
Total-seq B mouse anti-human CD122 (IL-2Rβ) (clone TU27; BioLegend; # 399904; PG; Human)
Total-seq B mouse anti-human FcεRIα (clone AER-37 (CRA-1); BioLegend; # 399904; PG; Human)
Total-seq B mouse anti-human CD41 (clone HIP8; BioLegend; # 399904; PG; Human)
Total-seq B mouse anti-human CD137 (4-1BB) (clone 4B4-1; BioLegend; # 399904; PG; Human)
Total-seq B mouse anti-human CD163 (clone GHI/61; BioLegend; # 399904; PG; Human)
Total-seq B mouse anti-human CD83 (clone HB15e; BioLegend; # 399904; PG; Human)
Total-seq B mouse anti-human CD124 (IL-4Rα) (clone G077F6; BioLegend; # 399904; PG; Human)
Total-seq B mouse anti-human CD13 (clone WM15; BioLegend; # 399904; PG; Human)
Total-seq B mouse anti-human CD2 (clone TS1/8; BioLegend; # 399904; PG; Human)
Total-seq B mouse anti-human CD226 (DNAM-1) (clone 11A8; BioLegend; # 399904; PG; Human)
Total-seq B mouse anti-human CD29 (clone TS2/16; BioLegend; # 399904; PG; Human)
Total-seq B mouse anti-human CD303 (BDCA-2) (clone 201A; BioLegend; # 399904; PG; Human)
Total-seq B mouse anti-human CD49b (clone P1E6-C5; BioLegend; # 399904; PG; Human)
Total-seq B mouse anti-human CD81 (TAPA-1) (clone 5A6; BioLegend; # 399904; PG; Human)
Total-seq B mouse anti-human IgD (clone IA6-2; BioLegend; # 399904; PG; Human)
Total-seq B mouse anti-human CD18 (clone TS1/18; BioLegend; # 399904; PG; Human)
Total-seq B mouse anti-human CD28 (clone CD28.2; BioLegend; # 399904; PG; Human)
Total-seq B mouse anti-human CD38 (clone HIT2; BioLegend; # 399904; PG; Human)
Total-seq B mouse anti-human CD127 (IL-7Rα) (clone A019D5; BioLegend; # 399904; PG; Human)
Total-seq B mouse anti-human CD45 (clone HI30; BioLegend; # 399904; PG; Human)
Total-seq B mouse anti-human CD22 (clone S-HCL-1; BioLegend; # 399904; PG; Human)
Total-seq B mouse anti-human CD71 (clone CY1G4; BioLegend; # 399904; PG; Human)
Total-seq B mouse anti-human CD26 (clone BA5b; BioLegend; # 399904; PG; Human)
Total-seq B mouse anti-human CD115 (CSF-1R) (clone 9-4D2-1E4; BioLegend; # 399904; PG; Human)
Total-seq B mouse anti-human CD63 (clone H5C6; BioLegend; # 399904; PG; Human)
Total-seq B mouse anti-human CD304 (Neuropilin-1) (clone 12C2; BioLegend; # 399904; PG; Human)
Total-seq B mouse anti-human CD36 (clone 5-271; BioLegend; # 399904; PG; Human)
Total-seq B mouse anti-human CD172a (SIRPα) (clone 15-414; BioLegend; # 399904; PG; Human)
Total-seq B mouse anti-human CD72 (clone 3F3; BioLegend; # 399904; PG; Human)
Total-seq B mouse anti-human CD158 (KIR2DL1/S1/S3/S5) (clone HP-MA4; BioLegend; # 399904; PG; Human)
Total-seq B mouse anti-human CD93 (clone VIMD2; BioLegend; # 399904; PG; Human)
Total-seq B mouse anti-human CD49a (clone TS2/7; BioLegend; # 399904; PG; Human)
Total-seq B mouse anti-human CD49d (clone 9F10; BioLegend; # 399904; PG; Human)
Total-seq B mouse anti-human CD73 (Ecto-5'-nucleotidase) (clone AD2; BioLegend; # 399904; PG; Human)
Total-seq B mouse anti-human CD9 (clone HI9a; BioLegend; # 399904; PG; Human)
Total-seq B mouse anti-human TCR Vα7.2 (clone 3C10; BioLegend; # 399904; PG; Human)

Total-seq B mouse anti-human TCR Vδ2 (clone B6; BioLegend; # 399904; PG; Human)
Total-seq B mouse anti-human LOX-1 (clone 15C4; BioLegend; # 399904; PG; Human)
Total-seq B mouse anti-human CD158b (KIR2DL2/L3, NKAT2) (clone DX27; BioLegend; # 399904; PG; Human)
Total-seq B mouse anti-human CD158e1 (KIR3DL1, NKB1) (clone DX9; BioLegend; # 399904; PG; Human)
Total-seq B mouse anti-human CD142 (clone NY2; BioLegend; # 399904; PG; Human)
Total-seq B mouse anti-human CD319 (CRACC) (clone 162.1; BioLegend; # 399904; PG; Human)
Total-seq B mouse anti-human CD352 (NTB-A) (clone NT-7; BioLegend; # 399904; PG; Human)
Total-seq B mouse anti-human CD94 (clone DX22; BioLegend; # 399904; PG; Human)
Total-seq B mouse anti-human CD162 (clone KPL-1; BioLegend; # 399904; PG; Human)
Total-seq B mouse anti-human CD85j (ILT2) (clone GHI/75; BioLegend; # 399904; PG; Human)
Total-seq B mouse anti-human CD23 (clone EBVCS-5; BioLegend; # 399904; PG; Human)
Total-seq B mouse anti-human CD328 (Siglec-7) (clone 6-434; BioLegend; # 399904; PG; Human)
Total-seq B mouse anti-human HLA-E (clone 3D12; BioLegend; # 399904; PG; Human)
Total-seq B mouse anti-human CD82 (clone ASL-24; BioLegend; # 399904; PG; Human)
Total-seq B mouse anti-human CD101 (BB27) (clone BB27; BioLegend; # 399904; PG; Human)
Total-seq B mouse anti-human CD88 (C5aR) (clone S5/1; BioLegend; # 399904; PG; Human)
Total-seq B mouse anti-human CD224 (clone KF29; BioLegend; # 399904; PG; Human)
Total-seq B mouse anti-human TIM3 (CD366) (clone F38-2E2; BioLegend; # 345053; PG; Human)
Total-seq B mouse anti-human CD99 (clone 3B2/TA8; BioLegend; # 371323; PG; Human)
Total-seq B mouse anti-human CD117 (clone 104D2; BioLegend; # 313247; PG; Human)
Total-seq B mouse anti-human CD34 (clone 581; BioLegend; # 343539; PG; Human)
Total-seq B mouse anti-human CD90 (clone 5E10; BioLegend; # 328147; PG; Human)
Total-seq B mouse anti-human CD10 (clone HI10a; BioLegend; # 312235; PG; Human, Cynomolgus, Rhesus)
Total-seq B mouse anti-human CD135 (clone BV10AH2; BioLegend; # 313321; PG; Human)
Total-seq B mouse anti-human CD371 (CLEC12A) (clone 50C1; BioLegend; # 353619; PG; Human)
Total-seq B mouse anti-human GPR56 (clone CG4; BioLegend; # 358211; PG; Human, Mouse)

M-FISH (clone; manufacturer; catalogue number; application; reactivity):
Goat anti-Avidin (clone -; Vector; # BA-0300; IF, ISH; All species)
Rabbit anti-Digoxin (polyclonal; Sigma Aldrich; # D7782; FISH, ChIP, AC)
Goat anti-rabbit Cy5.5 (polyclonal; Linaris, # PAK0027; IF, EB; Rabbit)
Streptavidin AF750 conjugate (clone -; Invitrogen; # S21384; IF; All species)

Validation

For all antibodies we relied on manufacturers' validation for species reactivity and applications. Validation data is available on the manufacturer's website with respective statements from manufacturer's websites also given above.

Animals and other research organisms

Policy information about studies involving animals; ARRIVE guidelines recommended for reporting animal research, and Sex and Gender in Research

Laboratory animals

Female NOD.Prkdcscid.Il2rgnull (NSG) mice 8-12 weeks of age were used in the study. Mice were housed in individually ventilated cages with controlled temperature (approx. 22 ºC) and humidity (50%) under 12-12 h light-dark cycle.

Wild animals

The study did not involve wild animals.

Reporting on sex

Findings apply only to one sex.

Field-collected samples

The study did not include samples collected from the field.

Ethics oversight

Animal experiments were conducted in compliance with all relevant ethical regulations. We obtained written, informed consent for all experiments and they were approved by the Regierungspräsidium Karlsruhe under Tierversuchsantrag numbers G42/18 and G-140-21.

Note that full information on the approval of the study protocol must also be provided in the manuscript.

Flow Cytometry

Plots

Confirm that:

☒ The axis labels state the marker and fluorochrome used (e.g. CD4-FITC).

☒ The axis scales are clearly visible. Include numbers along axes only for bottom left plot of group (a 'group' is an analysis of identical markers).

☒ All plots are contour plots with outliers or pseudocolor plots.

☒ A numerical value for number of cells or percentage (with statistics) is provided.

Methodology

Sample preparation

Primary human AML cells at diagnosis were recovered from cryopreserved bone marrow and/or peripheral blood samples. Patient-derived xenografts were generated by intrafemoral injection of 1-2 Million viable primary AML cells in NSG mice.

PDX-derived cells were frozen until processing. All samples were thawed at 37 °C in Iscove's modified Dulbecco's medium (IMDM) containing 10% FBS, and treated with DNase I for 15min (100 µg/ml).
For CITE-seq analysis, recovered cells were stained with a total of 38 or 149 antibody-derived tags (ADTs) as well as CD45, CD3 and 7-AAD (see Supplementary Table 11). Cells were sorted for live CD45+ cells.
For ex vivo drug profiling, recovered cells were cultured using previously established protocol using IMDM, 15% BIT (bovine serum albumin, insulin, transferrin; Stem Cell Technologies, cat # 09500), 100 ng/ml SCF (PeproTech, cat # 300-07), 50 ng/ml FLT3-L (PeproTech, cat # 300-19), 20 ng/ml IL-3 (PeproTech, cat # 200-03), 20 ng/ml G-CSF (PeproTech, cat # 300-23), 100 µM β-mercaptoethanol (ThermoFisher, cat # 31350010), 500 nM SR1 (StemRegenin 1, STEMCELL Technologies, cat # 72342), 500 nM UM729 (STEMCELL Technologies, cat # 72332), and 1% penicillin-streptomycin (Sigma, cat # P4458-100ML). 0.5x10^5 AML cells/well were seeded in flat-bottom 96-well plates, and cells were treated with up to 12 treatment conditions consisting of standard chemotherapy regimens as well as novel compounds for 24h and for selected conditions also for 72h (Supplementary Table 9). After 24h/72h, the cells were stained with cell surface antibodies (see Supplementary Table 12). Same amount of CountBright Absolute Counting Beads (Thermo Fisher Scientific, cat # C36950) together with 7-AAD (BD Biosciences, cat # 559925) were added to each sample prior to analysis with BD LSRFortessa Cell Analyzer.

Instrument

BD FACSAria™ Fusion I or II Cell Sorter, BD FACSAria™ III Cell Sorter, BD LSRFortessa™ Cell Analyzer

Software

FlowJo, BD FACSDiva

Cell population abundance

Due to limited sample material, post-sorting purities were not re-assessed using flow cytometry. Instead, this was done by gating and quantificatoin of populations using FlowJo. On average 80.75% of the total events were included after gating out debris in the FSC-A vs SSC-A plot; on average 98.7% of these events were within the single cells gate (based on FSC-A vs FSC-H); on average 89.8% of these single cells were gated as viable cells (based on 7-AAD vs SSC-A); and the final sorting population of CD45+ cells represented on average 99.7% of these viable cells (based on CD45 vs SSC-A).

Gating strategy

For CITE-seq sorting: FSC-A vs SSC-A was the starting gate wherein debris was excluded. Next, single cells were gated based on the exclusion of outliers in FSC-A vs FSC-H. Viable cells were then gated within this population based on low 7-AAD staining (SSC-A vs 7-AAD). Finally, the ultimate sorting population of CD45+ leukocytes were gated based on dim to high expression of CD45 (SSC-A vs CD45).
For ex vivo drug profiling: The gating strategy of live leukemic cells is depicted in Supplementary Figure 12. FSC-A vs SSC-A was the starting gate wherein debris was excluded. Next, single cells were gated based on the exclusion of outliers in FSC-A vs FSC-H. Viable cells were then gated within this population based on low 7-AAD staining (SSC-A vs 7-AAD). Next lineage-positive cells were excluded and leukemic cells were gated based on low expression of the lineage markers (CD3/CD20/CD235a vs CD45). Finally, leukemic cells were discriminated from the remaining immune cells via dim expression of CD45 and low to mid/high SSC-A (CD45 vs SSC-A).

☒ Tick this box to confirm that a figure exemplifying the gating strategy is provided in the Supplementary Information.
